# The Effects of Patient Education on Psychological Status and Clinical Outcomes in Rheumatoid Arthritis: A Systematic Review and Meta-Analysis

**DOI:** 10.3389/fpsyt.2022.848427

**Published:** 2022-03-17

**Authors:** Zugui Wu, Yue Zhu, Yi Wang, Rui Zhou, Xiangling Ye, Zehua Chen, Congcong Li, Junyi Li, Zixuan Ye, Zhenbang Wang, Wengang Liu, Xuemeng Xu

**Affiliations:** ^1^The Fifth Clinical Medical College, Guangzhou University of Chinese Medicine, Guangzhou, China; ^2^Baishui Health Center, Qujing, China; ^3^Guangzhou University of Chinese Medicine, Guangzhou, China; ^4^Qujing Hospital of Traditional Chinese Medicine, Qujing, China; ^5^Guangdong Second Traditional Chinese Medicine Hospital, Guangzhou, China

**Keywords:** patient education, psychotherapy, rheumatoid arthritis, meta-analysis, systematic review

## Abstract

**Background:**

Rheumatoid arthritis (RA) is a common systemic inflammatory autoimmune disease. The disease has a serious impact on mental health and requires more effective non-pharmacological interventions.

**Objective:**

This study aims to systematically evaluate the effectiveness of patient education on psychological status and clinical outcomes in rheumatoid arthritis.

**Methods:**

This systematic review and meta-analysis was conducted based on the Preferred Reporting Items for Systematic Reviews and Meta-Analyses (PRISMA) guidelines. PubMed, Cochrane Library, EMBASE database, and Web of Science database were screened for articles published until November 2, 2021. Randomized controlled trials (RCTs) of patient education for RA were included. Outcomes measures included pain, physical function, disease activity, erythrocyte sedimentation rate (ESR), C-reactive protein (CRP), anxiety, depression, Arthritis Self-Efficacy (pain, other symptoms, total), and General health. For each outcome, standardized mean differences or mean differences and 95% confidence intervals (CIs) were calculated.

**Results:**

A total of 24 RCTs (*n* = 2,276) were included according to the inclusion and exclusion criteria. Meta-analysis revealed a statistically significant overall effect in favor of patient education for physical function [SMD = −0.52, 95% CI (−0.96, −0.08), *I*^2^ = 93%, *P* = 0.02], disease activity [SMD = −1.97, 95% CI (−3.24, −0.71), *I*^2^ = 97%, *P* = 0.002], ASE (pain) [SMD = −1.24, 95% CI (−2.05, −0.43), *I*^2^ = 95%, *P* = 0.003], ASE (other symptoms) [SMD = −0.25, 95% CI (−0.41, −0.09), *I*^2^ = 25%, *P* = 0.002], ASE (total) [SMD = −0.67, 95% CI (−1.30, −0.05), *I*^2^ = 90%, *P* = 0.03], and general health [SMD = −1.11, 95% CI (−1.36, −0.86), *I*^2^ = 96%, P < 0.00001]. No effects were found for anxiety [SMD = 0.17, 95% CI (−0.64, 0.98), *I*^2^ = 82%, *P* = 0.68], depression [SMD = −0.18, 95% CI (−0.52, 0.15), *I*^2^ = 52%, *P* = 0.28], pain [SMD = −0.37, 95% CI (−0.80, 0.05), *I*^2^ = 89%, *P* = 0.08], and CRP [SMD = −0.27, 95% CI (−0.57, 0.02), *I*^2^ = 0%, *P* = 0.07].

**Conclusions:**

Patient education may be effective in improving clinical outcomes and psychological status in patients with rheumatoid arthritis. Considering the methodological limitations of the included RCTs, more high-quality and large-sample RCTs are needed to confirm this conclusion in the future.

**Systematic Review Registration:**

http://www.crd.york.ac.uk/prospero, identifier: CRD42021250607.

## Introduction

Rheumatoid arthritis (RA) is an autoimmune disease characterized by chronic inflammation, which can cause joint destruction, deformity, pain, and dysfunction (1, 2). This chronic disease has led to a decline in patient's physical function, quality of life, and workability, as well as an increase in medical expenses (3, 4), which brings a heavy burden to individuals and society (5). At present, RA has become one of the main global public health problems, affecting nearly 1% of the world's population (6). RA is clinically incurable, but antirheumatic drugs and biological agents can control symptoms and improve inflammation (7). However, the efficacy of drugs was affected by patients' adherence with medications (8), and oral medications have brought many adverse reactions to patients (9). Studies have found that about 12%-17% of patients have adverse drug events after discharge (10). Therefore, doctors and patients are often looking for more beneficial non-pharmacological interventions. In addition, some studies have found that 30 to 80% of patients with rheumatic and chronic musculoskeletal diseases do not adhere to treatment plans, that poor patient adherence to treatment can affect treatment outcomes, and that poor adherence is associated with reduced functioning and health-related quality of life (11–13). Therefore, strategies to improve patient adherence are critical to improving the effectiveness of clinical interventions (14). Several studies have made recommendations to increase patient adherence, such as patient-centredness, the inclusion of patients in treatment decisions, and patient participation in shared decision-making may be critical factors in improving adherence (13). Some studies suggest that individualized patient education improves patient adherence (15, 16).

Patient education is a low-cost intervention with no side effects, and it has been accepted by patients, family members, and medical workers. At present, the educational intervention has become an effective supplement to traditional medical treatment, which aims to support and help patients with RA to strengthen their life and health management (17). Previous studies have found that educational interventions can increase awareness of patients with RA about the disease and treatment methods, thereby improving their medication adherence (17, 18). Other studies have reported that educational intervention may have a positive effect on the control of disease activity (19, 20). At the same time, educational intervention can improve the health, pain, swollen joint count, tender joint count, and physical function of patients with RA (21). However, other studies have shown that the effect of educational intervention on disease control was not yet clear, the short-term and long-term effects may be inconsistent (22, 23).

According to previously reported studies, the effectiveness of patient education interventions is still controversial. It is necessary to conduct a systematic review and meta-analysis to evaluate its efficacy. Although some previous studies have summarized the effect of patient education on rheumatoid arthritis using a systematic review approach, this study did not perform a meta-analysis (24, 25). Another meta-analysis summarized the impact of patient education on rheumatoid arthritis, reporting outcomes including disability, tender joint count, depression, general health, and psychological status. The study found that patient education had a short-term effect on rheumatoid arthritis and no long-term effects. The literature included in this study was mainly published before 2001, which was published a long time ago (26). In recent years, due to the growing interest of researchers in patient education, many randomized controlled trials of patient education in the treatment of rheumatoid arthritis have been published. It is necessary to recapitulate and update this evidence based on the latest published literature. This meta-analysis aims to review and analyze the effectiveness of patient education in the treatment of RA. Several variables were compared, including pain, physical function, disease activity, erythrocyte sedimentation rate (ESR), C-reactive protein (CRP), anxiety, depression, Arthritis Self-Efficacy (pain, other symptoms, total), and general health.

## Methods

This study was conducted according to the PRISMA guidelines and the recommendations of the Cochrane Collaboration (27). All analyses were based on previously published studies, and ethical approval was not required in this review. Systematic Review Registration: http://www.crd.york.ac.uk/prospero, identifier: CRD42021250607.

### Search Strategy

We searched all clinical studies published in PubMed, Cochrane Library, Embase, and Web of Science database before November 2, 2021. Search terms such as the following were used: “Arthritis, Rheumatoid,” “Rheumatoid Arthritis,” “Education,” “Educational Activities,” “Training Programs,” “Workshops,” “Randomized Controlled Trial,” “Clinical Trial,” “Randomly,” “Randomised,” and “random allocation.” We conducted a detailed search for each database according to the search methods of the different databases. The Supplementary Appendix described the search strategy of each database in detail. Four researchers screened the retrieved documents according to the inclusion criteria and exclusion criteria to read the title, abstract, and full text. The disagreements between the four researchers were discussed with the fifth researcher until a consensus was reached.

### Selection Criteria

#### Patients

This study included patients with RA, and the diagnostic criteria included diagnosis based on doctors (Physician diagnosed), or the diagnostic criteria of the American College of Rheumatology (ACR). There were no restrictions on the patients' age, gender, course of the disease, and where RA occurs.

#### Interventions

Patient education is defined as a planned and systematic educational activity aimed at improving the health of patients, such as providing disease-related information, health consultation, behavior guidance, behavior modification and advice (24, 28). These methods are aimed at improving the patient's experience of their disease, raising awareness of the disease, promoting the patient's healthy behavior, and improving the patient's ability to deal with the disease. These educational activities can be carried out verbally, in writing, or remotely (such as by telephone). Patient education for patients with rheumatoid arthritis mainly includes providing patients with rheumatoid arthritis with disease-related information, treatment methods, coping strategies for disease symptoms, suggestions and guidance on daily activities, and other activities to improve patients' disease knowledge and health behavior. These educational activities are planned and systematic. We also excluded studies that systematically taught patients to exercise or exercise therapy with the primary goal of exercise or increasing their exercise adherence. However, some suggestions for exercise methods can be used as part of the intervention component of patient education. There were no restrictions on the duration, frequency, and specific methods of educational intervention.

#### Comparisons

In this study, the types of interventions in the intervention and control group included education vs. usual care, education + usual care vs. usual care, education + conventional treatment vs. conventional treatment, and education vs. waiting list (no intervention). Conventional treatment includes other treatments that patients used before entering the study, such as medications or other routine treatments. No intervention mainly refers to that the control group patients were in the waiting list group and did not receive any intervention before the end of the study, while the same interventions as the intervention group were used after the end of the study.

#### Outcomes

For inclusion in this review, RCTs had to assess at least one outcome, and the outcome parameters in the respective studies had to be the primary outcomes:

1. Pain. Pain was measured using the visual analog scale (VAS), or Arthritis Impact Measurement Scales 2 (AIMS 2).
2. Physical function. Physical function was measured using the Health Assessment Questionnaire (HAQ), Arthritis Impact Measurement Scales 2 (AIMS2), or the Short-Form Health Survey (SF-36).
3. Disease activity. Disease activity was measured using the disease activity score 28 (DAS-28) or Ritchie Articular Index (RAI).
4. Erythrocyte sedimentation rate (ESR).
5. C-reactive protein (CRP).
6. Anxiety. Anxiety was measured using the Hospital Anxiety and Depression Scale (HADS) or State-Trait Anxiety Inventory (STAI).
7. Depression. Depression was measured using the Hospital Anxiety and Depression Scale (HADS), Beck Depression Inventory (BDI), or Center for Epidemiologic Studies Depression Scale (CES-D).
8. Arthritis Self-Efficacy (pain, other symptoms, total). ASE was measured using the Arthritis Self-Efficacy scale (ASE).
9. General health. General health was measured using the Short-Form Health Survey (SF-36), EuroQol Five Dimensions Questionnaire (EQ5D), or Arthritis Impact Measurement Scales 2 (AIMS2).

#### Studies Types

This study included only randomized controlled trials (RCTs). Observational studies, non-randomized controlled trials, and reviews were not included. The language of the included studies was limited to English.

#### Data Extraction and Quality Assessment

Four reviewers independently extracted study data from eligible studies according to a prespecified study protocol, including the characteristics of the researchers (e.g., name and country), patient characteristics (e.g., age, gender, and duration of disease), research characteristics (e.g., study design, publication years, sample size, the frequency and duration of intervention), and study outcomes. When the follow-up time was inconsistent between studies, we chose to include the final follow-up time. The disagreements between the four researchers were discussed with the fifth researcher until a consensus was reached.

#### Assessment of Risk of Bias in Included Studies

Two reviewers independently used the Cochrane risk-of-bias tool to assess the quality and risk of bias of the included studies, which included the following domains: selection bias (random sequence generation and allocation concealment), performance bias (blinding of participants and personnel), detection bias (blinding of outcome assessment), attrition bias (incomplete outcome data), reporting bias (selective reporting), and other bias (29). The evaluation results were examined by a third reviewer, and the disagreed evaluations were further discussed until a consensus was reached.

#### Rating Quality of Evidence

The Grading of Recommendations, Assessment, Development and Evaluation (GRADE) system was used to evaluate the quality of evidence for each outcome. The strength of the evidence was categorized as high, moderate, low, or very low. Two reviewers independently used the GRADE system to assess the quality of evidence. The disagreements between the two researchers were discussed with the third researcher until a consensus was reached.

### Statistical Analysis

We conducted this meta-analysis of the included literature by Review Manager 5.3 software (Cochrane Collaboration, Oxford, UK) and illustrated the results of data merging intuitively with a forest map. The mean differences (MDs), standard mean differences (SMDs), and 95% confidence intervals (CIs) were calculated by random-effects models or fixed-effects models. The heterogeneity between various studies was statistically analyzed by *I*^2^ and chi-square tests. Significant heterogeneity was indicated when *I*^2^ ≥ 50% or *P* < 0.1, and the random-effects models were used. When *I*^2^ < 50% or *P* > 0.1 showed no significant heterogeneity, the fixed-effects models were used. When there was heterogeneity among various studies, a subgroup analysis was conducted according to the type of interventions. We used meta-regression and sensitivity analysis to explore the sources of heterogeneity. In addition, publication bias was assessed using Egger's and Begg's tests (30). *P*-values < 0.05 were considered statistically significant.

## Results

### Study Selection

**Figure 1:**
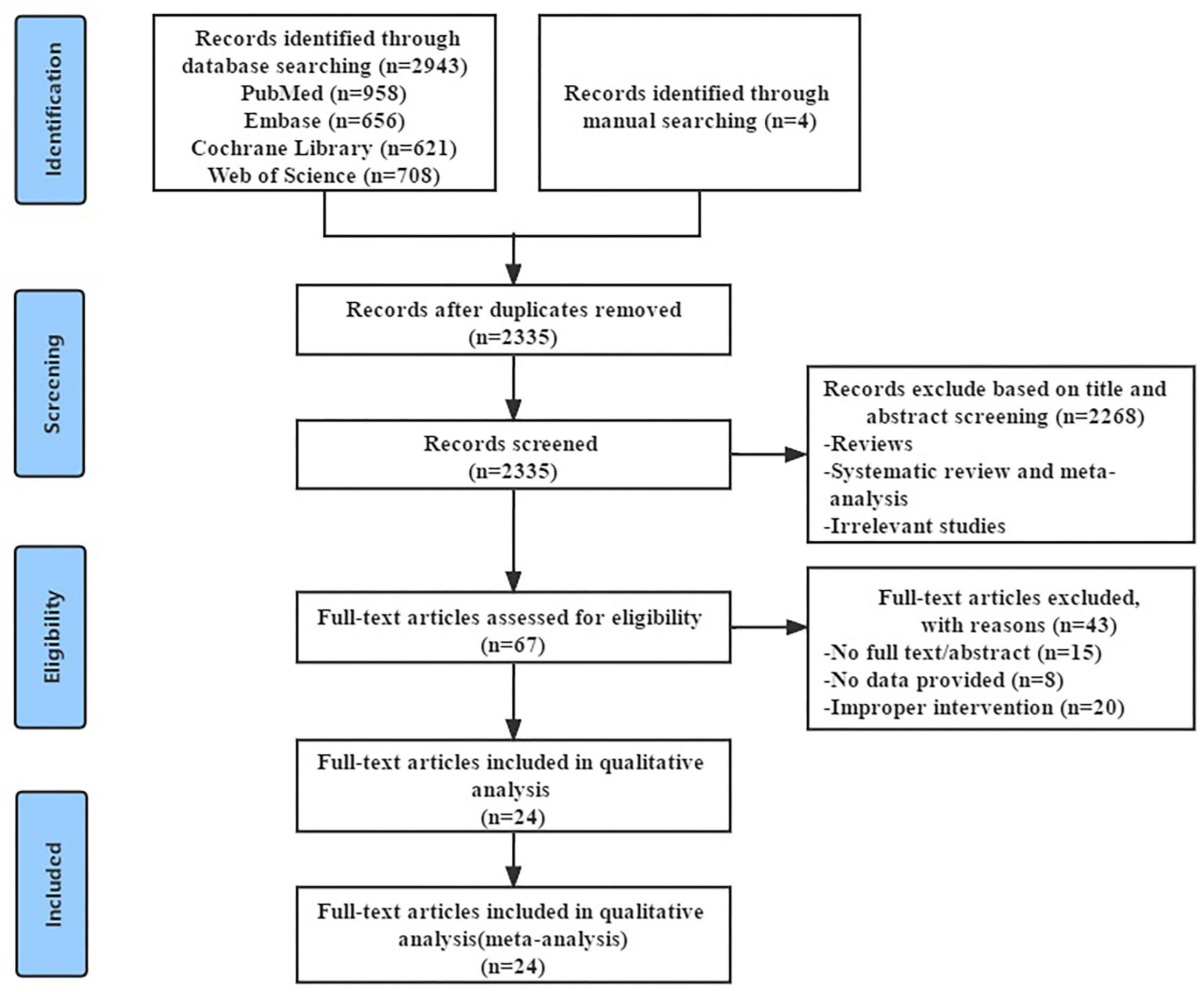
Flowchart of meta-analysis search and selection process.

The literature search strategy was detailed in the Supplementary Appendix, and the screening process was summarized in Figure 1. We searched the four English databases (PubMed, Cochrane Library, Embase, and Web of Science database). Initially, we retrieved a total of 2,947 potentially relevant records and excluded 612 duplicate records. After reading the title and abstract, we excluded 2,268 obviously irrelevant records, leaving 67 research need to read the full text for further confirmation. After reading the full text, 43 studies that did not meet the inclusion criteria were excluded, 24 RCTs were retained. Finally, 24 RCTs (31–54) were included, with a total of 2,276 patients with RA.

### Study Characteristics

#### Overview of Included Studies

The study characteristics of the 24 RCTs can be observed in Table 1. These studies were published from 1991 to 2021. The 24 RCTs included were conducted in different countries, with two in the United States (44, 45), three in France (40, 43, 47), four in China (51–54), four in United Kingdom (32, 34, 36, 37), four in Iran (46, 48–50), one each in the Canada (31), Austria (33), Netherlands (35) Spain (38), Italy (39), Brazil (41), and Australia (42). Of the 24 studies included, 2,276 patients with RA were analyzed. The number of patients with RA in each study ranged from 32 to 258, with a total of 1,155 patients in the education group and 1,121 in the control group. Twenty-four studies reported the age of patients with RA. Their mean age ranged from 44.27 to 69.03 years. Eighteen studies reported the duration of symptoms, with the mean duration ranged from 7.05 to 20.48 years. Sixteen studies were based on the diagnosis of KOA based on clinical and radiographic features by a physician (33–35, 37–44, 46, 47, 49, 51, 53), while the remaining eight studies were based on the diagnosis criteria of the American College of Rheumatology (31, 32, 36, 45, 48, 50, 52, 54).

**Table 1:** Study characteristics.

**References**	**Population**	**Country**	**Years**	**Study design**	**Mean age (SD), years**		**Sample size**		**Male/female**		**Symptom duration in years (SD)**	
					**EG**	**CG**	**EG**	**CG**	**EG**	**CG**	**EG**	**CG**
Helewa et al. (31)	RA^c^	Canada	1991	RCT	52.7 (12.6)	55.3 (11.8)	53	52	Not reported		Not reported	
Barlow et al. (32)	RA^c^	The U.K.	1997	RCT	58.62 (11.25)	60.04 (10.82)	53	55	9/44	11/44	14.62 (11.49)	17.04 (12.29)
Scholten et al. (33)	RA^b^	Austria	1999	RCT	48.3 (5.6)		38	30	14/54		8.9 (1.2)	
Hill et al. (34)	RA^b^	The U.K.	2001	RCT	63 (Not reported)	62 (Not reported)	51	49	17/34	10/39	13 (Not reported)	12 (Not reported)
Riemsma et al. (35)	RA^b^	Netherlands	2003	RCT	55.1 (10.3)	57.0 (8.3)	71	76	24/47	29/47	11.7 (11.1)	11.4 (8.9)
Hammond et al. (36)	RA^c^	The U.K.	2004	RCT	53.9 (13.9)	57.1 (13.5)	162	164	41/121	49/115	9.0 (7.7)	9.9 (8.8)
Kirwan et al. (37)	RA^b^	The U.K.	2005	RCT	56.4 (10.17)	57.1 (10.71)	30	28	11/19	7/21	13.2 (12.72)	16.7 (12.18)
Montserrat et al. (38)	RA^b^	Spain	2006	RCT	55.40 (16.32)	51.09 (16.62)	22	21	14/8	15/6	21.50 (15.30)	19.47 (16.09)
Masiero et al. (39)	RA^b^	Italy	2007	RCT	54.2 (9.8)	52.5 (11.9)	36	34	7/29	6/28	14.8 (8.8)	16.1 (8.3)
Giraudet-Le Quintrec et al. (40)	RA^b^	France	2007	RCT	55.32 (11.08)	54.31 (14.37)	104	104	15/89	16/88	11.85 (9.44)	14.25 (10.27)
Lovisi et al. (41)	RA^b^	Brazil	2009	RCT	45.71 (10.50)	46.20 (9.52)	28	30	2/26	5/25	9.43 (9.10)	9.41 (7.95)
Macedo et al. (42)	RA^b^	Australia	2009	RCT	48.63 (11.56)	52.56 (7.65)	16	16	1/15	1/15	11.63 (9.95)	8.38
Mathieux et al. (43)	RA^b^	France	2009	RCT	48.3 (13.0)	47.0 (13.2)	30	30	8/22	9/21	Not reported	
Conn et al. (44)	RA^b^	The U.S.	2013	RCT	54.2 (8.2)	52.9 (10.2)	52	52	11/41	11/41	9.1 (Not reported)	6.4 (Not reported)
Shigaki et al. (45)	RA^c^	The U.S.	2013	RCT	50.3 (11.6)	49.3 (12.3)	54	54	4/50	6/48	7.4 (8.6)	8.5 (10.3)
Yousefi et al. (46)	RA^b^	Iran	2015	RCT	42.9 (13.24)	46.6 (10.97)	100	106	14/86	11/95	7.6 (5.18)	6.51 (5.28)
Pot-Vaucel et al. (47)	RA^d^	France	2016	RCT	58.2 (10.7)	62.4 (9.8)	28	26	Not reported		11.6 (9.4)	14.5 (3.0)
Anvar et al. (48)	RA^c^	Iran	2018	RCT	69.03 (Not reported)		37	39	0/37	0/39	Not reported	
Hosseini Moghadam et al. (49)	RA^a^	Iran	2018	RCT	48.06 (10.51)	48.87 (9.24)	32	32	Not reported	Not reported		
Saeedifar et al. (50)	RA^c^	Iran	2018	RCT	44.27 (11.35)		30	30	Not reported		9.1 (Not reported)	
Zhao et al. (51)	RA^a^	China	2019	RCT	56.93 (11.14)	54.15 (10.06)	46	46	12/34	14/32	Not reported	
Shao et al. (52)	RA^c^	China	2020	RCT	60.40 (12.04)	56.59 (7.12)	15	17	5/10	4/13	9.57 (9.74)	10.88 (7.32)
Song et al. (53)	RA^a^	China	2020	RCT	57.05 (11.31)	53.22 (10.04)	41	36	11/30	11/25	Not reported	
Shao et al. (54)	RA^c^	China	2021	RCT	58.2 (11.3)	59.5 (11.9)	112	112	18/94	14/98	10.2 (8.1)	11.1 (8.9)

*^a^2010 ACR/EULAR Diagnostic criteria; ^b^1987ACR Diagnostic criteria; ^c^Physician diagnosed RA; RA^d^, 2009 ACR Diagnostic criteria. U.S., United States of America; U.K., United Kingdom; RCT, Randomized Controlled Trials; SD, Standard deviation; EG, Education group; CG, Control group*.

#### Intervention Characteristics and Outcome Measures

Table 2 shows the characteristics of interventions in these 24 RCTs, including the specific methods of education, duration of intervention, and outcomes. To compare interventions between the education group and the control group, eight studies used education vs. usual care (32, 35, 37, 44, 48, 51, 52, 54), and five studies used education + usual care vs. usual care (36, 40, 42, 46, 53), five studies used education + conventional treatment vs. conventional treatment (34, 38, 39, 49, 50), and six studies used education vs. waiting list (no intervention) (31, 33, 41, 43, 45, 47). The outcomes of these 24 RCTs include pain, physical function, disease activity, erythrocyte sedimentation rate (ESR), C-reactive protein (CRP), anxiety, depression, ASE (pain), ASE (other symptoms), ASE(total), and general health. Twelve studies that assessed pain used VAS (10 cm) scores (32, 34, 37, 38, 41, 50),VAS (100 mm) scores (36, 39, 42, 46), and AIMS2 scores (35, 45), respectively. Fifteen studies assessed physical function using HAQ (31, 33, 36–44, 51, 54), SF-36 (46) and AIMS2 (35), respectively. Eight studies assessed disease activity using DAS-28 (35, 40, 42, 51–54) and RAI (39), respectively. Five studies assessed the anxiety using HADS (32, 37, 40) and STAI (41, 47), respectively. Five studies assessed the depression using HADS (32, 37, 40), BDI (31, 33, 41, 47), and CES-D (45), respectively. Ten studies assessed ASE using Arthritis Self-Efficacy Questionnaire (32, 35–37, 45, 48, 49, 51, 52, 54). Four studies assessed the general health using SF-36 (41, 46), AIMS2 (45) and EQ5D (42), respectively.

**Table 2:** Intervention, main measures, and results.

**References**	**Specific methods of education**	**Intervention length, frequency, and duration**		**Main outcomes and results**
		**Education group**	**Control group**	
Helewa et al. (31)	Patients were provided with joint protection education, daily living skills and coping strategies	Education (6 weeks)	Waiting list	1. Physical function (HAQ); 2. Depression (Beck)
Barlow et al. (32)	Patients were educated through leaflets that include information about rheumatoid arthritis disease, disease management and medication	Education (3 weeks)	Usual care	1. Pain (VAS-10 cm); 2. Anxiety (HADS); 3. Depression (HADS); 4. ASE (pain); 5. ASE (other symptoms)
Scholten et al. (33)	Multidisciplinary education of patients through lectures provides information on disease and medication, pain management and joint protection, and coping skills for disease symptoms and sequelae	Education (6 weeks)	Waiting list	1. Physical function (HAQ); 2. Depression (BDI)
Hill et al. (34)	Educational curricula have been developed for patients, including information on rheumatoid arthritis, treatment medications, pain management strategies, joint protection skills, exercise advice and other daily coping strategies	Education (30 min each; 7 times in total; 24 weeks) + Conventional treatment	Conventional treatment	1. Pain (VAS-10 cm); 2. CRP
Riemsma et al. (35)	Group education was used to provide educational courses for patients, and education-related pamphlets and tapes were distributed to provide patients with information about diseases and treatments, as well as various coping strategies related to diseases, so as to promote patients to strengthen the management of diseases	Education (2.5-h each; Five times a week; 6 weeks)	Usual care	1. Pain (AIMS); 2. Physical function (AIMS); 3. ASE (pain); 4. ASE (other symptoms); 5. Disease activity (DAS-28)
Hammond et al. (36)	Basic information on rheumatoid arthritis and its treatment and management, coping strategies for daily life, exercise and advice on joint protection were provided to patients through education	Education (1 times per week; 5 weeks) + Usual care	Usual care	1. Pain (VAS-100 mm); 2. Physical function (HAQ); 3. ASE (total)
Kirwan et al. (37)	Education was provided to patients through courses, including knowledge of the disease, advice and guidance on coping strategies in daily life, pain management, emotional management, joint protection, drug use, and so on	Education (2.5-h each; The first, second, third, fourth and eighth weeks; Five times in total)	Usual care	1. Pain (VAS-10 cm); 2. Physical function (HAQ); 3. Anxiety (HADS); 4. Depression (HADS); 5. ASE (pain); 6. ASE (other symptoms)
Montserrat et al. (38)	Through a combination of individual and group education, rheumatoid arthritis knowledge, joint protection methods, exercise advice and pain management strategies were taught to patients	Education (30 min each; Once every 3 months; 12 weeks) + Conventional treatment	Conventional treatment	1. Pain (VAS-10 cm); 2. Physical function (HAQ); 3. ESR; 4. CRP
Masiero et al. (39)	Through lectures, conferences and pamphlets, patients were taught about the disease, the mechanism of pain and other symptoms, as well as information about exercise and pain management	Education (6-h each; Once every 3 weeks; 12 weeks) + Conventional treatment	Conventional treatment	1. Pain (VAS-100 mm); 2. Physical function (HAQ); 3. Disease activity (RAI)
Giraudet-Le Quintrec et al. (40)	Through multidisciplinary education, patients were taught information about the disease, diet, exercise and treatment methods, as well as coping strategies for certain diseases	Education (6-h each; Eight times a week; 72weeks) + Usual care	Usual care	1. Physical function (HAQ); 2. Anxiety (HADS); 3. Depression (HADS); 4. Disease activity (DAS-28)
Lovisi et al. (41)	Comprehensive information on rheumatoid arthritis, including etiology, pathogenesis, disease management, drug therapy and rehabilitation, was taught to patients through meetings	Education (1-h each; Once a week; 6 weeks)	Waiting list	1. Pain (VAS-10 cm); 2. Physical function (HAQ); 3. Anxiety (STAI); 4. Depression (BDI); 5. General health (SF36)
Macedo et al. (42)	Provide patients with information about rheumatoid arthritis, medications, and coping strategies for their daily lives	Education (1 times per week; 5 weeks) + Usual care	Usual care	1. Pain (VAS-100 mm); 2. Physical function (HAQ); 3. Disease activity (DAS-28); 4. General health (EQ5D); 5. ESR
Mathieux et al. (43)	Provide multidisciplinary education to patients, including information on disease and treatment, advice and guidance on joint protection	Education (24 weeks)	Waiting list	1. Physical function (HAQ)
Conn et al. (44)	Provide educational manuals for patients to increase their understanding of the disease and treatment, provide coping strategies for the disease, and increase patients' management of the disease	Education (2-h each; 1 times per week; 6 weeks)	Usual care	1. Physical function (HAQ)
Shigaki et al. (45)	Provide educational courses for patients, including the causes of illness, treatment of illness, and pain management	Education (6 weeks)	Waiting list	1. Pain (AIMS2); 2. Depression (CES-D); 3. ASE (total); 4. General health (AIMS2)
Yousefi et al. (46)	Provide multidisciplinary education and educational brochures to patients to increase their awareness of rheumatoid arthritis, provide pain management methods and joint protection methods, and enhance patients' disease management skills	Education (5-h each; 1 times per week; 6 weeks) + Usual care	Usual care	1. Pain (VAS-100 mm); 2. Physical function (SF36); 3. General health (SF36)
Pot-Vaucel et al. (47)	To provide patients with information about the disease and treatment, and to increase their understanding of the disease and treatment, and to provide disease management skills and medication information	Education (24 weeks)	Waiting list	1. Anxiety (STAI); 2. Depression (Beck)
Anvar et al. (48)	A multidisciplinary group education approach was used to improve patient awareness of all aspects of the disease, coping strategies for pain management and daily activities, and information on diet and exercise	Education (1–1.5 h each; 6 times per week; 6 weeks)	Usual care	1. ASE (pain);
Hosseini Moghadam et al. (49)	Group education for patients with rheumatoid arthritis includes providing coping strategies for the disease, guidance on daily activities and precautions to improve their knowledge of RA	Education (30 min each; Twice a week; 8 weeks) + Conventional treatment	Conventional treatment	1. ASE (pain); 2. ASE (other symptoms); 3. ASE (total)
Saeedifar et al. (50)	Patients were provided with information about rheumatoid arthritis, joint protection methods, pain management strategies, methods to prevent a recurrence, appropriate exercise patterns, and enhanced management of the disease	Education (1–2 h each; 1 times per week; 24 weeks) + Conventional treatment	Conventional treatment	1. Pain (VAS-10 cm)
Zhao et al. (51)	The clinical guidelines on rheumatoid arthritis were taught by telephone, including basic information on rheumatoid arthritis, as well as medication, diet and exercise	Education (20–40 min each; Once every 3 weeks; 12 weeks)	Usual care	1. Physical function (HAQ); 2. ASE (total); 3. Disease activity (DAS-28)
Shao et al. (52)	Through the course, patients will be provided with disease-related knowledge and information, coping skills and joint protection strategies to enhance patients' management of disease	Education (6 weeks)	Usual care	1. Disease activity (DAS-28); 2. ASE (pain); 3. ASE (other symptoms)
Song et al. (53)	Educational courses were provided to patients over the phone, which included knowledge and skills in rheumatoid arthritis, treatment methods, information on medication, exercise and diet	Education (20–40 min each; Once every 3 weeks; 12 weeks) + Usual care	Usual care	1. Disease activity (DAS-28); 2. ESR
Shao et al. (54)	Through the course, patients are provided with disease-related knowledge and information, disease-related coping skills and joint protection strategies to enhance their disease management	Education (25–40 min a day, 8 weeks)	Usual care	1. Physical function (HAQ); 2.ASE (pain); 3. ASE (other symptoms); 4. Disease activity (DAS-28)

*HAQ, Health Assessment Questionnaire; AIMS2, Arthritis Impact Measurement Scales 2; SF36, The Short-Form Health Survey; DAS28, Disease Activity Score 28; RAI, Ritchie Articular Index; HADS, Hospital Anxiety and Depression Scale; STAI, State-Trait Anxiety Inventory; BDI, Beck Depression Inventory; CES-D, Center for Epidemiologic Studies Depression Scale; ASE, Arthritis Self-Efficacy Questionnaire; VAS, Visual analogue scale; EQ5D, EuroQol Five Dimensions Questionnaire*.

#### Risk of Bias

**Figure 2:**
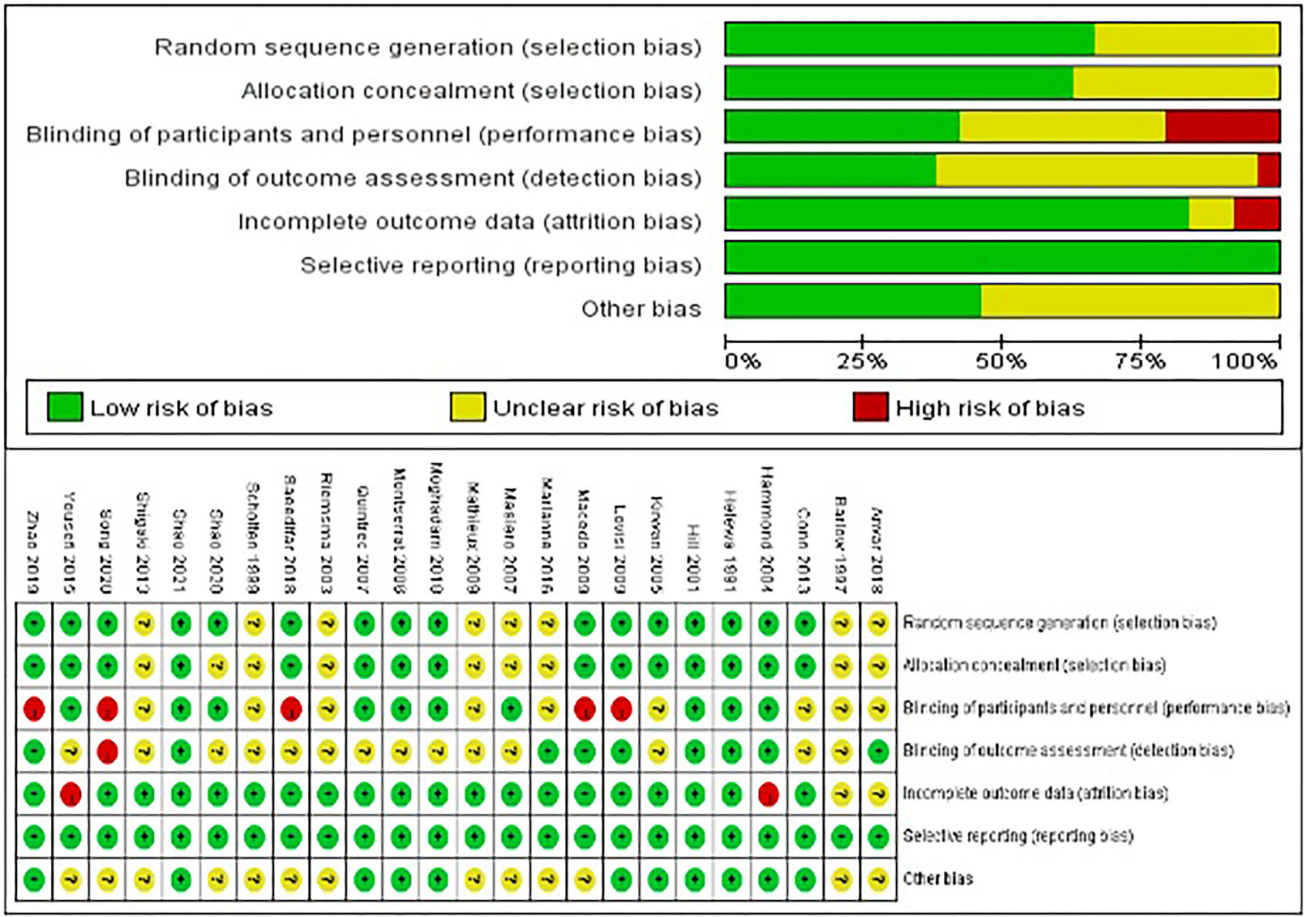
Risk of bias graph.

The details of the risk of bias for each study can be observed in Figure 2. Sixteen studies reported specific randomization methods (31, 34, 36–38, 40–42, 44, 46, 50–54), eight RCTs did not specify the specific randomization method (32, 33, 35, 39, 43, 45, 47, 48). The allocation concealment of nine RCTs remained unclear (32, 33, 35, 38, 43, 45, 47, 48, 52). The performance bias of five RCTs was judged to be high (41, 42, 50, 51, 53), and nine RCTs was judged to be unclear (32, 33, 35, 37, 43–45, 47, 48). The detection bias of one RCT was judged to be high (53), and fourteen RCTs remained unclear (32, 33, 35, 37–40, 43–46, 49, 50, 52). Two RCT was judged to be high in incomplete outcome data (attrition bias) (36, 46), and two RCTs remained unclear (32, 48). The other bias of the thirteen studies was unclear (32, 33, 35, 39, 42, 43, 45–48, 50, 52, 53). Among the twenty-four RCTs included, only three RCTs had a low risk of publication bias (31, 34, 54).

#### Quality of Evidence

The results of the quality of evidence assessed by the GRADE system can be found in Table 3. The quality of evidence for pain, physical function, disease activity, and depression was classified as moderate. The quality of evidence for anxiety, ASE (pain), ASE (other symptoms), and general health was classified as low. The quality of evidence for ESR, CRP, and ASE (total) was classified as very low.

**Table 3:** Evidence quality rated using the GRADE approach.

**Outcomes**	**No. of studies**	**Sample size**	**Risk of bias**	**Inconsistency**	**Indirectness**	**Imprecision**	**Publication bias**	**Evidence quality**	
Pain	12	1,160	Not serious	Serious	Not serious	Not serious	Not serious	⊕⊕⊕⊖	Moderate
Physical function	15	1,641	Not serious	Serious	Not serious	Not serious	Not serious	⊕⊕⊕⊖	Moderate
Disease activity	8	838	Not serious	Serious	Not serious	Not serious	Not serious	⊕⊕⊕⊖	Moderate
ESR	3	152	Serious	Not serious	Not serious	Very serious	Not serious	⊕⊖⊖⊖	Very low
CRP	3	220	Serious	Not serious	Not serious	Very serious	Not serious	⊕⊖⊖⊖	Very low
Anxiety	5	443	Not serious	Serious	Not serious	Serious	Not serious	⊕⊕⊖⊖	Low
Depression	8	700	Not serious	Serious	Not serious	Not serious	Not serious	⊕⊕⊕⊖	Moderate
ASE (pain)	7	675	Serious	Serious	Not serious	Not serious	Not serious	⊕⊕⊖⊖	Low
ASE (other symptoms)	6	599	Serious	Serious	Not serious	Not serious	Not serious	⊕⊕⊖⊖	Low
ASE (total)	4	487	Serious	Serious	Not serious	Serious	Not serious	⊕⊖⊖⊖	Very low
General health	4	340	Serious	Not serious	Not serious	Serious	Not serious	⊕⊕⊖⊖	Low

*ESR, erythrocyte sedimentation rate; CRP, C-reactive protein; ASE, Arthritis Self-Efficacy*.

### Assessment of Overall Effect Size

#### Pain

**Figure 3:**
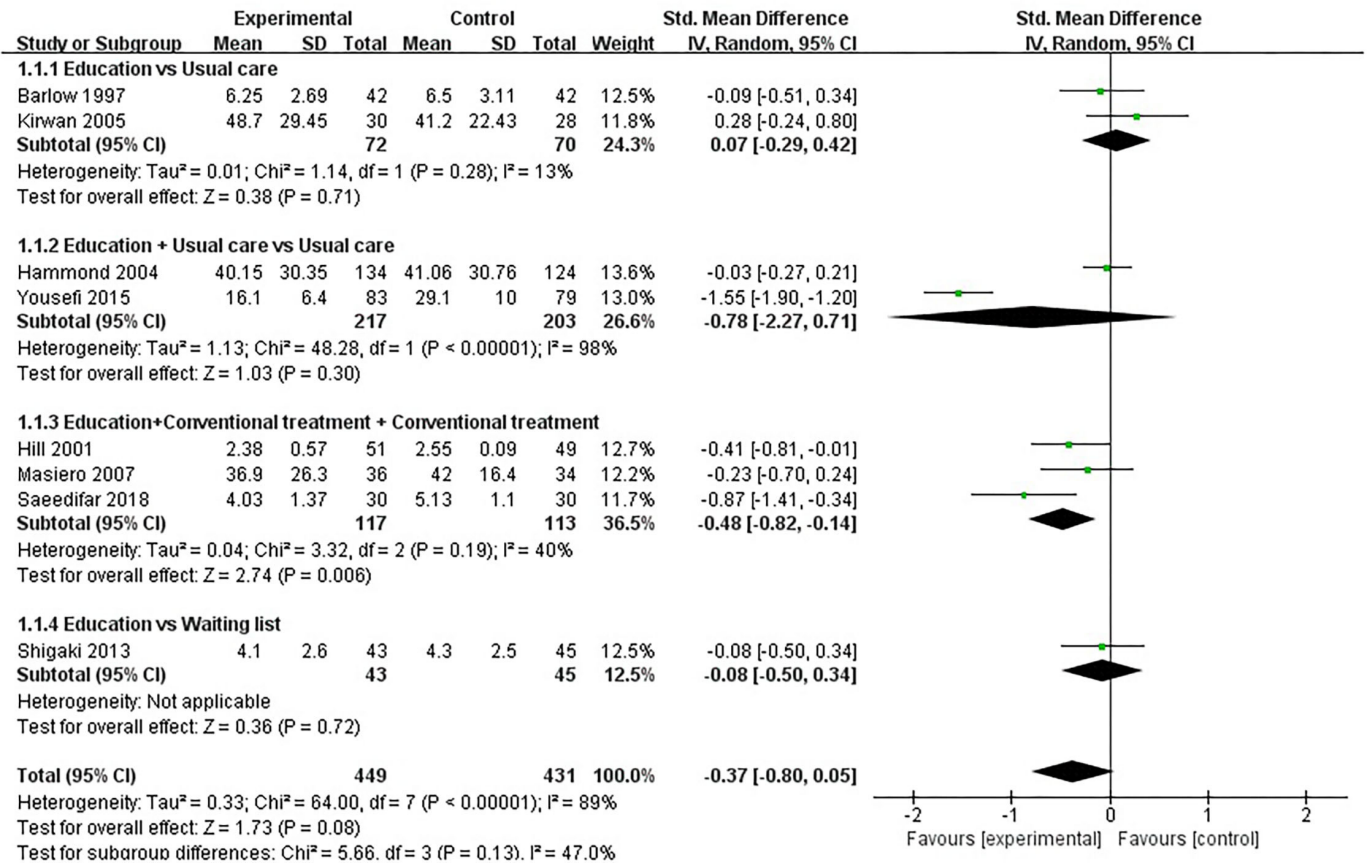
Meta-analysis on Pain.

Twelve studies evaluated pain and included 1,160 participants. Twelve studies that assessed pain used VAS (10 cm) scores (32, 34, 37, 38, 41, 50), VAS (100 mm) scores (36, 39, 42, 46), and AIMS2 scores (35, 45), respectively. The lower the VAS score, and AIMS2 score, the less painful. Of the 12 RCTs, five studies reported that patient education improved pain in patients with RA (*P* < 0.05) (38, 42, 46, 50), while the other seven studies reported that patient education did not improve pain in patients with RA (*P* > 0.05) (32, 34–37, 39, 41, 45). Four RCTs was not included in the meta-analysis (35, 38, 41, 42). Eight of the twelve RCTs were included in the meta-analysis (32, 34, 36, 37, 39, 45, 46, 50). The pooled results showed no significant improvement in pain in the education group compared to the control group [SMD = −0.37, 95% CI (−0.80, 0.05), *I*^2^ = 89%, *P* = 0.08]. When education was compared with usual care, the subgroup analysis showed no significant difference in pain improvement in the education group [SMD = 0.07, 95% CI (−0.29, 0.42), *I*^2^= 13%, *P* = 0.71]. Similarly, when education + usual care was compared with usual care alone, subgroup analysis showed no difference in pain improvement in the education group [SMD = −0.78, 95% CI (−2.27, 0.71), *I*^2^ = 98%, *P* = 0.30]. In addition, when education was compared with waiting list (no intervention), subgroup analysis showed no difference in pain improvement in the education group [SMD = −0.08, 95% CI (−0.50, 0.34), *P* = 0.72]. However, when education + conventional treatment was compared with conventional treatment alone, subgroup analysis showed a significant difference in pain improvement in the education group [SMD = −0.48, 95% CI (−0.82, −0.14), *I*^2^ = 40%, *P* = 0.006] (Figure 3).

#### Physical Function

**Figure 4:**
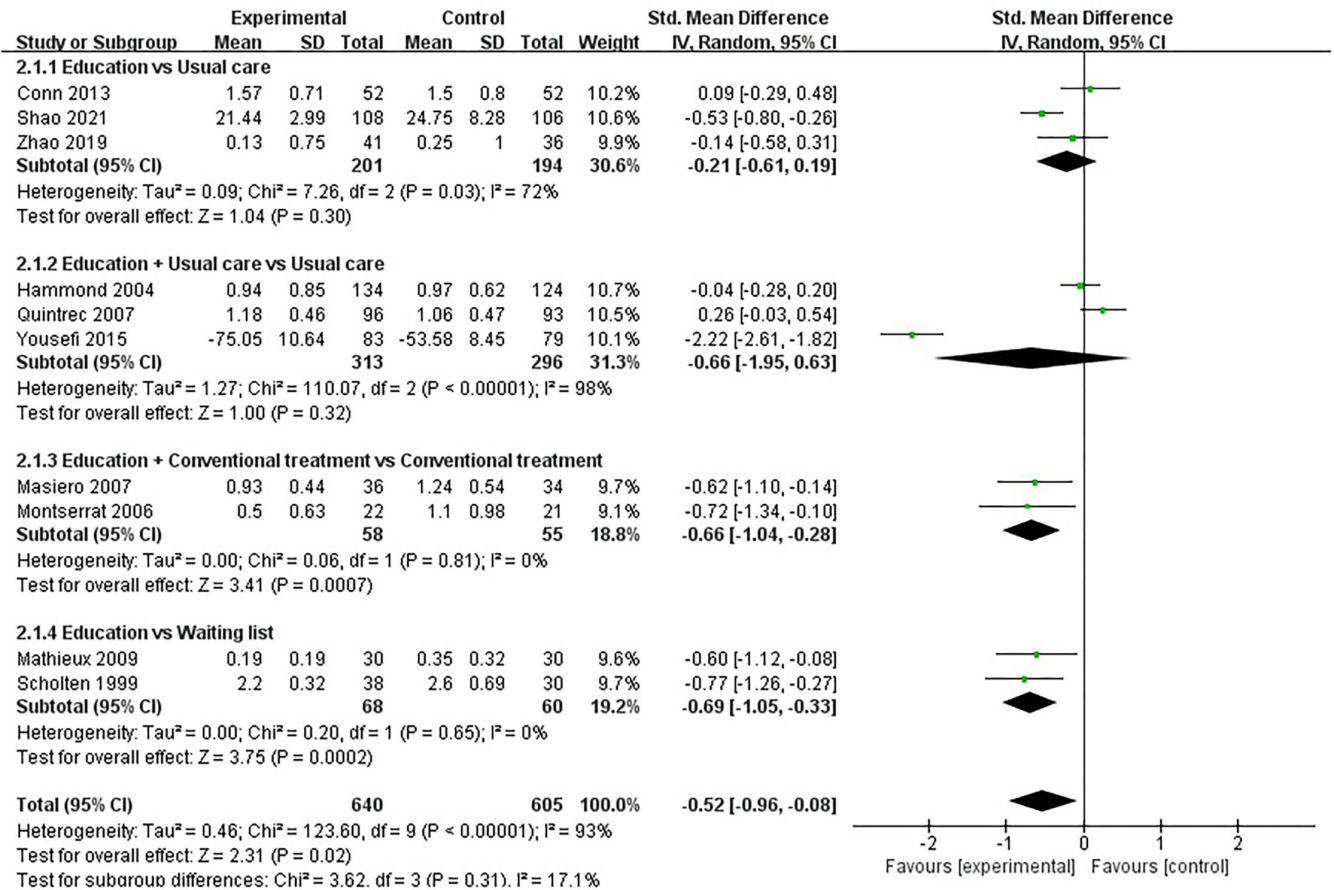
Meta-analysis on Physical function.

Physical function was assessed in fifteen studies involving 1,641 participants. Fifteen studies assessed physical function using HAQ (31, 33, 36–44, 51, 54), SF-36 (46) and AIMS2 (35), respectively. The lower the HAQ score and AIMS2 score, the better the physical function, while the higher the SF-36 score, the better the physical function. Of the fifteen RCTs, six studies reported that patient education improved physical function in patients with RA (*P* < 0.05) (33, 38, 39, 43, 46, 54), while the other nine studies reported that patient education did not improve physical function in patients with RA (*P* > 0.05) (31, 35–37, 40–42, 44, 51). Five RCTs was not included in the meta-analysis (31, 35, 37, 41, 42). Ten of the 15 RCTs were included in the meta-analysis (33, 36, 38–40, 43, 44, 46, 51, 54). Pooled results showed a significant improvement in physical function in the education group compared to the control group [SMD = −0.52, 95% CI (−0.96, −0.08), *I*^2^ = 93%, *P* = 0.02]. When education was compared with usual care, the subgroup analysis showed no significant difference in physical function improvement in the education group [SMD = −0.21, 95% CI (−0.61, 0.19), *I*^2^= 72%, *P* = 0.30]. Similarly, when education + usual care was compared with usual care alone, subgroup analysis showed no difference in physical function improvement in the education group [SMD = −0.66, 95% CI (−1.95, 0.63), *I*^2^ = 98%, *P* = 0.32]. However, when education was compared with waiting list (no intervention), subgroup analysis showed a significant difference in physical function improvement in the education group [SMD = −0.69, 95% CI (−1.05, −0.33), *I*^2^ = 0%, *P* = 0.0002]. In addition, when education + conventional treatment was compared with conventional treatment alone, subgroup analysis showed a significant difference in physical function improvement in the education group [SMD = −0.66, 95% CI (−1.04, −0.28), *I*^2^ = 0%, *P* = 0.0007] (Figure 4).

#### Disease Activity

**Figure 5:**
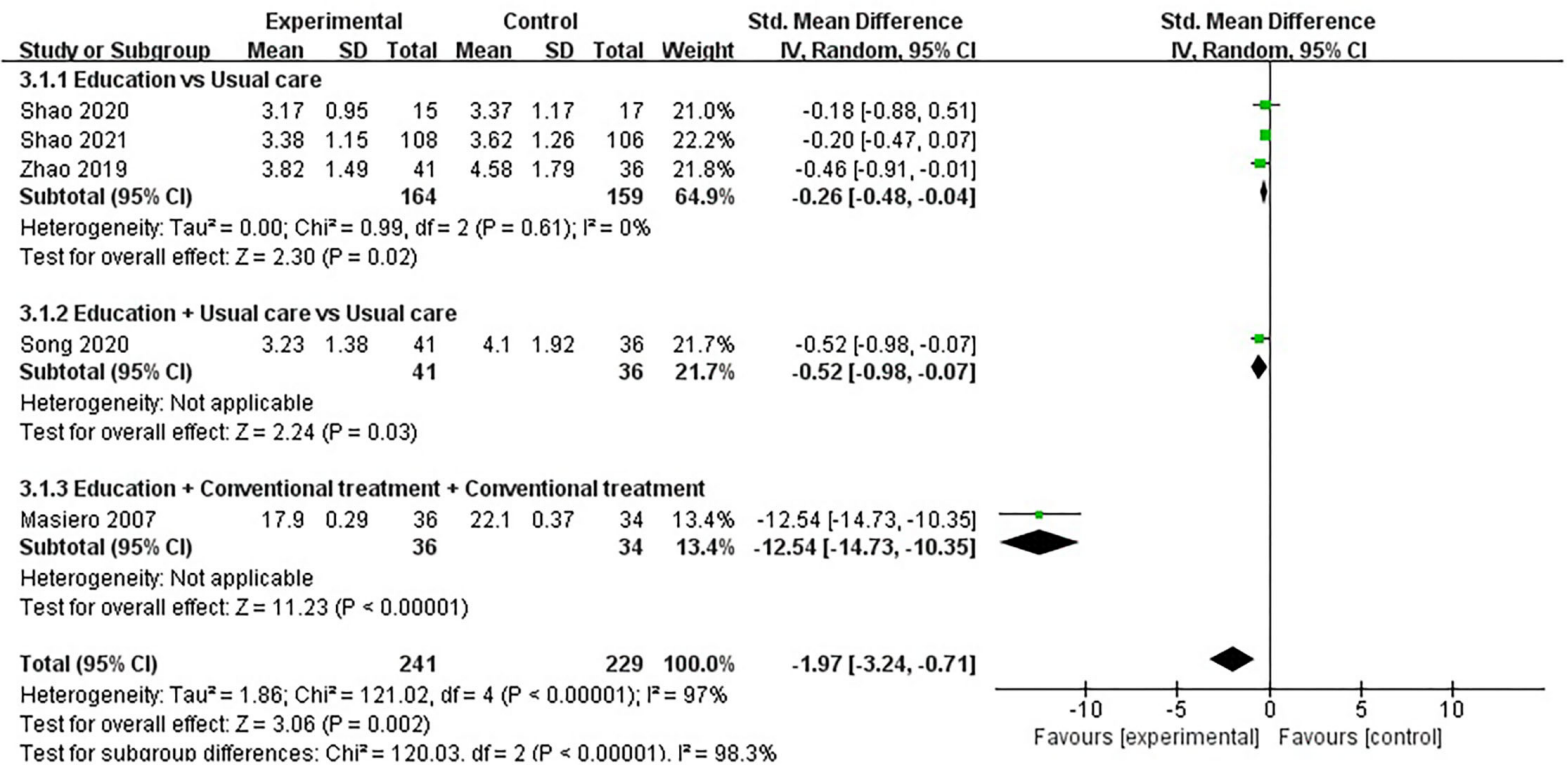
Meta-analysis on Disease activity.

Eight studies assessed disease activity, involving a total of 838 participants. Eight studies assessed disease activity using DAS-28 (35, 40, 42, 51–54) and RAI (39), respectively. The lower the DAS-28 score and RAI score, the better the disease activity. Of the eight RCTs, four studies reported that patient education improved disease activity in patients with RA (*P* < 0.05) (35, 38, 51, 53), while the other four studies reported that patient education did not improve disease activity in patients with RA (*P* > 0.05) (40, 42, 52, 54). Three RCTs was not included in the meta-analysis (35, 40, 42). Five of the eight RCTs were included in the meta-analysis (39, 51–54). Pooled results showed a significant improvement in disease activity in the education group compared to the control group [SMD = −1.97, 95% CI (−3.24, −0.71), *I*^2^ = 97%, *P* = 0.002]. When education was compared with usual care, subgroup analysis showed that the education group had significantly improved disease activity [SMD = −0.26, 95% CI (−0.48, −0.04), *I*^2^ = 0%, *P* = 0.02]. Similarly, when education + usual care was compared with usual care alone, subgroup analysis showed that the education group had significantly improved disease activity [SMD = −0.52, 95% CI (−0.98, −0.07), *P* = 0.03]. In addition, when education + conventional treatment was compared with conventional treatment alone, subgroup analysis showed a significant difference in disease activity improvement in the education group [SMD = −12.54, 95% CI (−14.73, −10.35), *P* < 0.00001] (Figure 5).

#### ESR

ESR were reported in three RCTs (38, 42, 53) with a total of 152 patients with RA. Three RCTs was not included in the meta-analysis (38, 42, 53). In three RCTs, the comparisons involved (1) education + conventional treatment was compared with conventional treatment alone, and (2) education + usual care was compared with usual care alone. When education + conventional treatment was compared with conventional treatment alone, the results showed that there was difference in improved ESR (*P* < 0.05). However, when education + usual care was compared with usual care alone, the results showed that there was no difference in improved ESR (*P* > 0.05).

#### CRP

**Figure 6:**
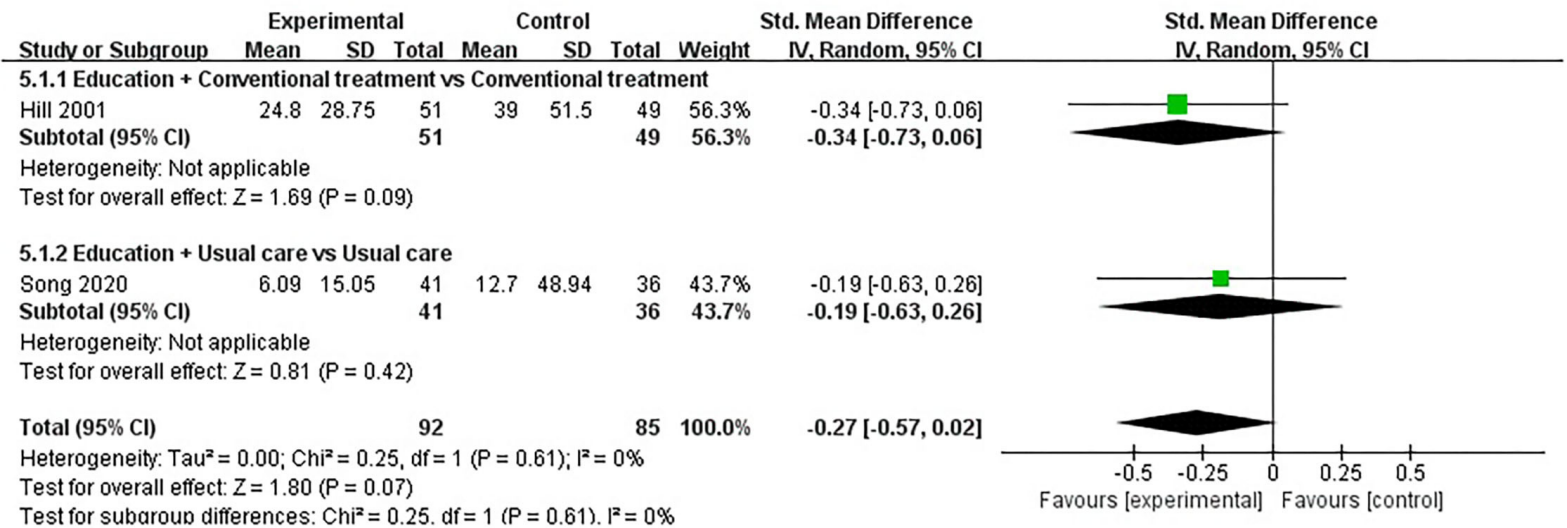
Meta-analysis on CRP.

Three studies assessed CRP and included a total of 220 participants. Of the three RCTs, three studies reported that patient education did not improve CRP in patients with RA (*P* > 0.05) (34, 38, 53). One RCT was not included in the meta-analysis (38). Two of the three RCTs were included in the meta-analysis (34, 53). The pooled results showed no significant improvement in CRP in the education group compared to the control group [SMD = −0.27, 95% CI (−0.57, 0.02), *I*^2^ = 0%, *P* = 0.07]. When education + conventional treatment was compared with conventional treatment alone, the subgroup analysis revealed that the education group showed no significant difference in improved CRP [SMD = −0.34, 95% CI (−0.73, 0.06), *P* = 0.09]. Similarly, when education was compared with usual care, subgroup analysis also showed no statistical difference in CRP improvement between the education group and usual care [SMD = −0.19, 95% CI (−0.63, 0.26), *P* = 0.42] (Figure 6).

#### Anxiety

**Figure 7:**
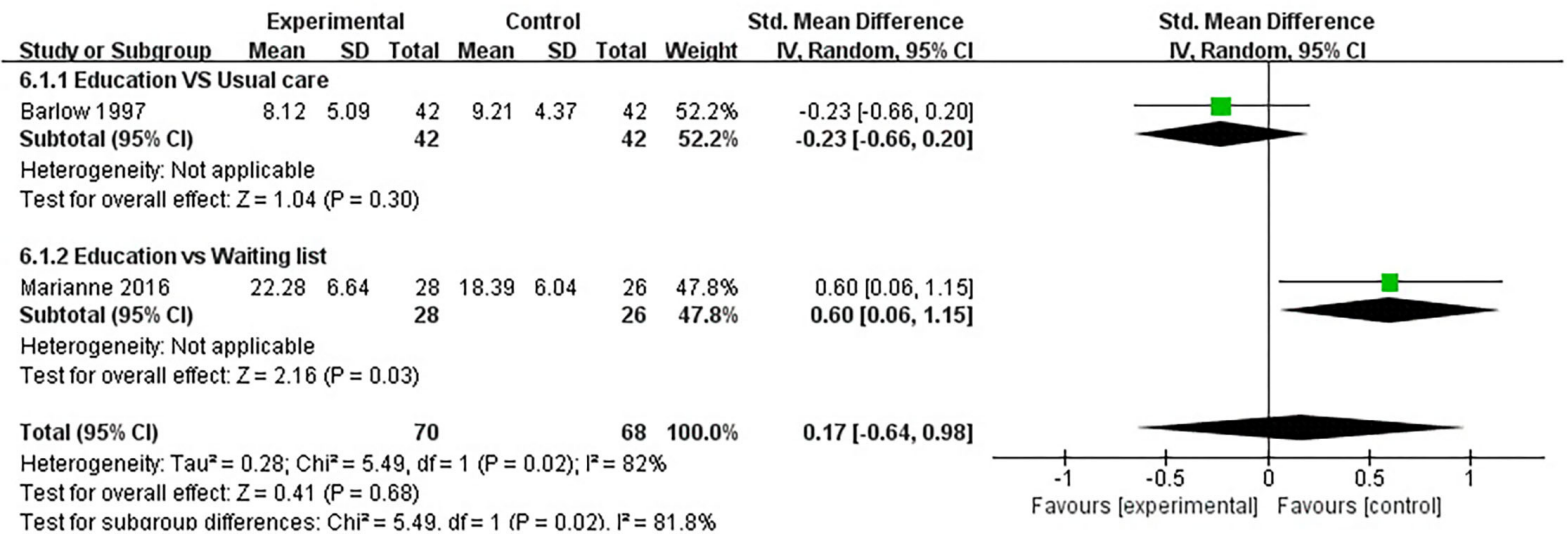
Meta-analysis on Anxiety.

Anxiety was evaluated in five studies, and 443 participants were included. Five studies assessed anxiety using HADS (32, 37, 40) and STAI (41, 47), respectively. The lower the HADS score and STAI score, the better the anxiety. Of the five RCTs, five studies reported that patient education did not improve anxiety in patients with RA (*P* > 0.05) (32, 37, 40, 41, 47). Three RCTs was not included in the meta-analysis (37, 40, 41). Two of the five RCTs were included in the meta-analysis (32, 47). The pooled results showed no significant improvement in anxiety in the education group compared to the control group [SMD = 0.17, 95% CI (−0.64, 0.98), *I*^2^ = 82%, *P* = 0.68]. When education was compared with usual care, the subgroup analysis showed no significant improvement in the anxiety of the education group [SMD = −0.23, 95% CI (−0.66, 0.20), *P* = 0.30]. However, when education was compared with waiting list (no intervention), the subgroup analysis revealed that the control group showed a significant difference in improved anxiety [SMD = 0.60, 95% CI (0.06, 1.15), *P* = 0.03] (Figure 7).

#### Depression

**Figure 8:**
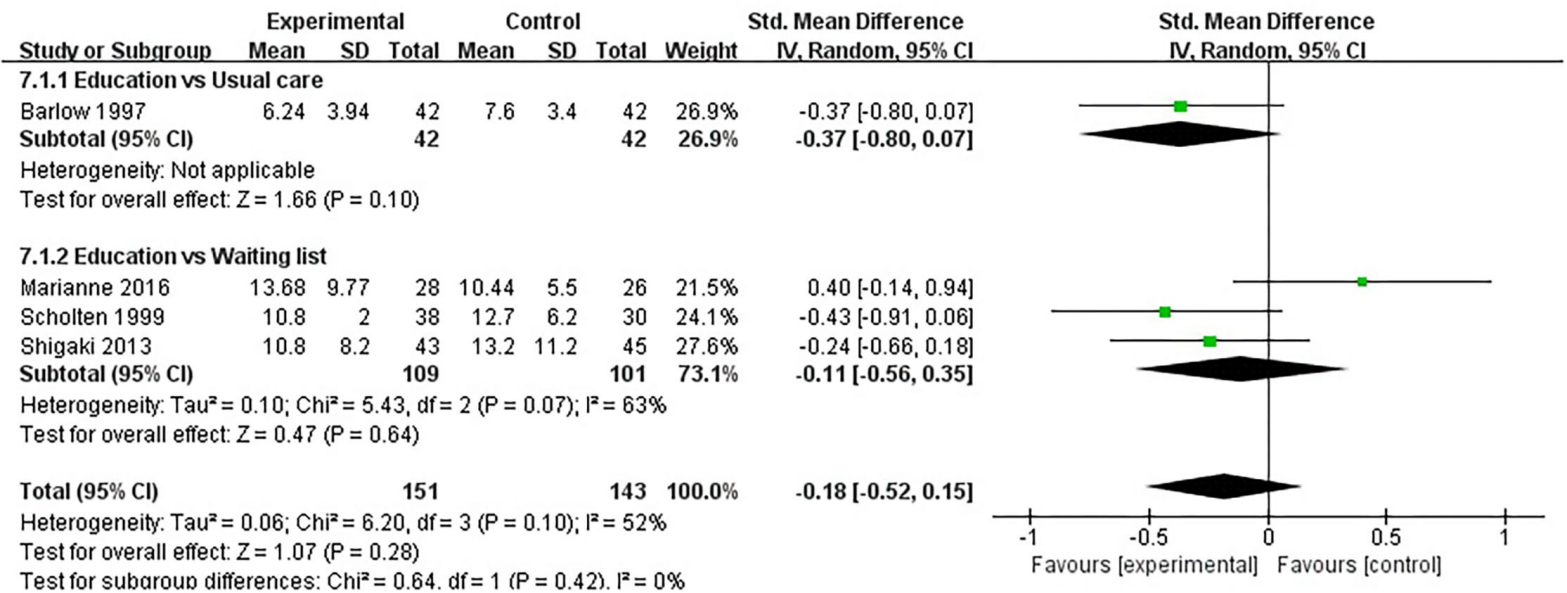
Meta-analysis on Depression.

Depression was evaluated in eight studies, and 700 participants were included. Eight studies assessed depression using HADS (32, 37, 40), BDI (31, 33, 41, 47), or CES-D (45), respectively. The lower the HADS score, BDI score and CES-D score, the better the depression. Of the eight RCTs, eight studies reported that patient education did not improve depression in patients with RA (P > 0.05) (31–33, 37, 40, 41, 45, 47). Four RCTs was not included in the meta-analysis (31, 37, 40, 41). Four of the eight RCTs were included in the meta-analysis (32, 33, 45, 47). The pooled results showed no significant improvement in depression in the education group compared to the control group [SMD = −0.18, 95% CI (−0.52, 0.15), *I*^2^ = 52%, *P* = 0.28]. When education was compared with usual care, the subgroup analysis showed no significant improvement in the depression of the education group [SMD = −0.37, 95% CI (−0.80, 0.07), *P* = 0.10]. In addition, When education was compared with waiting list (no intervention), the subgroup analysis showed that there was no significant difference in improved depression [SMD = −0.11, 95% CI (−0.56, 0.35), *I*^2^ = 63%, *P* = 0.64] (Figure 8).

#### ASE (Pain)

**Figure 9:**
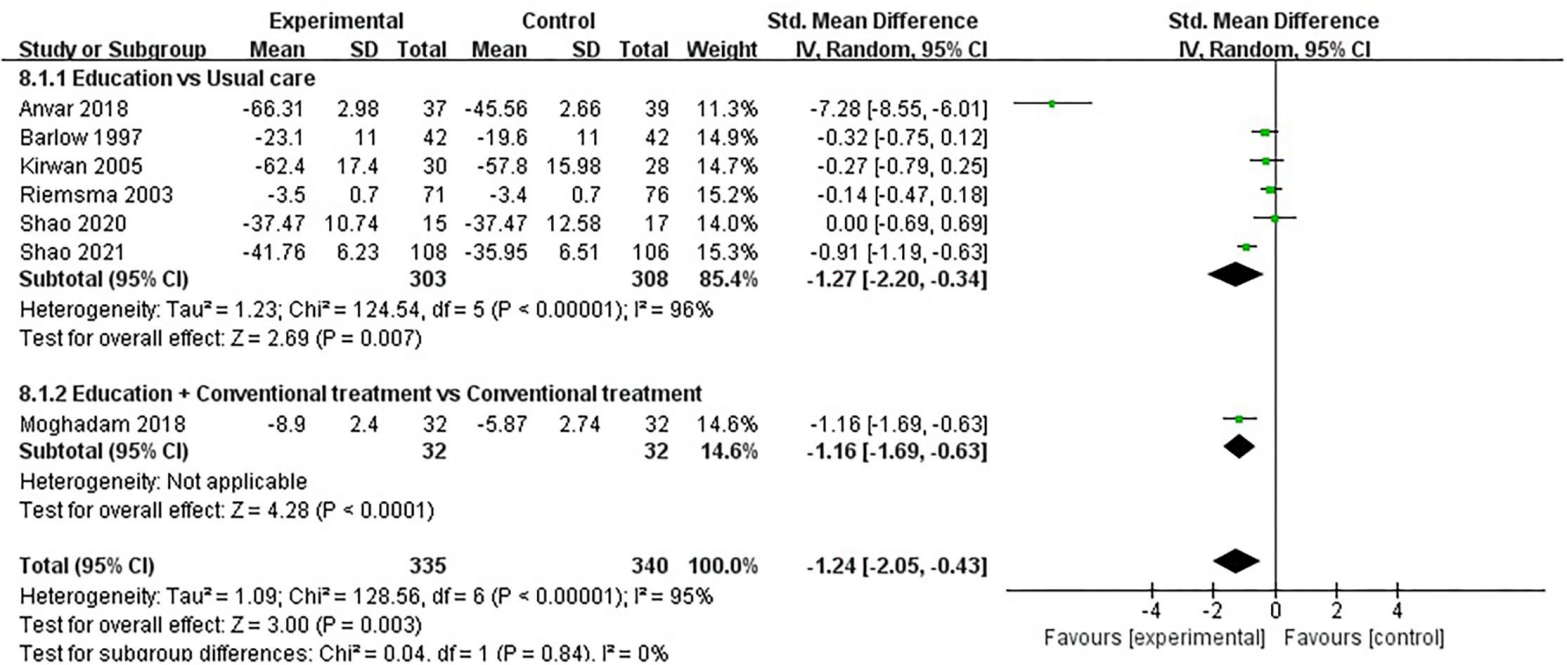
Meta-analysis on ASE (pain).

ASE (pain) was evaluated in seven studies involving a total of 675 participants. Seven studies assessed ASE (pain) using the ASE scale (32, 35, 37, 48, 49, 52, 54). Of the seven RCTs, three studies reported that patient education improved ASE (pain) in patients with RA (*P* < 0.05) (48, 49, 54), while the other four studies reported that patient education did not improve ASE (pain) in patients with RA (*P* > 0.05) (32, 35, 37, 52). Seven RCTs were included in the meta-analysis (32, 35, 37, 48, 49, 52, 54). Pooled results showed a significant improvement in ASE(pain) in the education group compared to the control group [SMD = −1.24, 95% CI (−2.05, −0.43), *I*^2^ = 95%, *P* = 0.003]. When education was compared with usual care, the subgroup analysis showed a significant improvement in ASE (pain) in the education group [SMD = −1.27, 95% CI (−2.20, −0.34), *I*^2^ = 96%, *P* = 0.007]. Similarly, when education + conventional treatment was compared with conventional treatment alone, subgroup analysis showed a significant improvement in ASE (pain) [SMD = −1.16, 95% CI (−1.69, −0.63), *P* < 0.0001] (Figure 9).

#### ASE (Other Symptoms)

**Figure 10:**
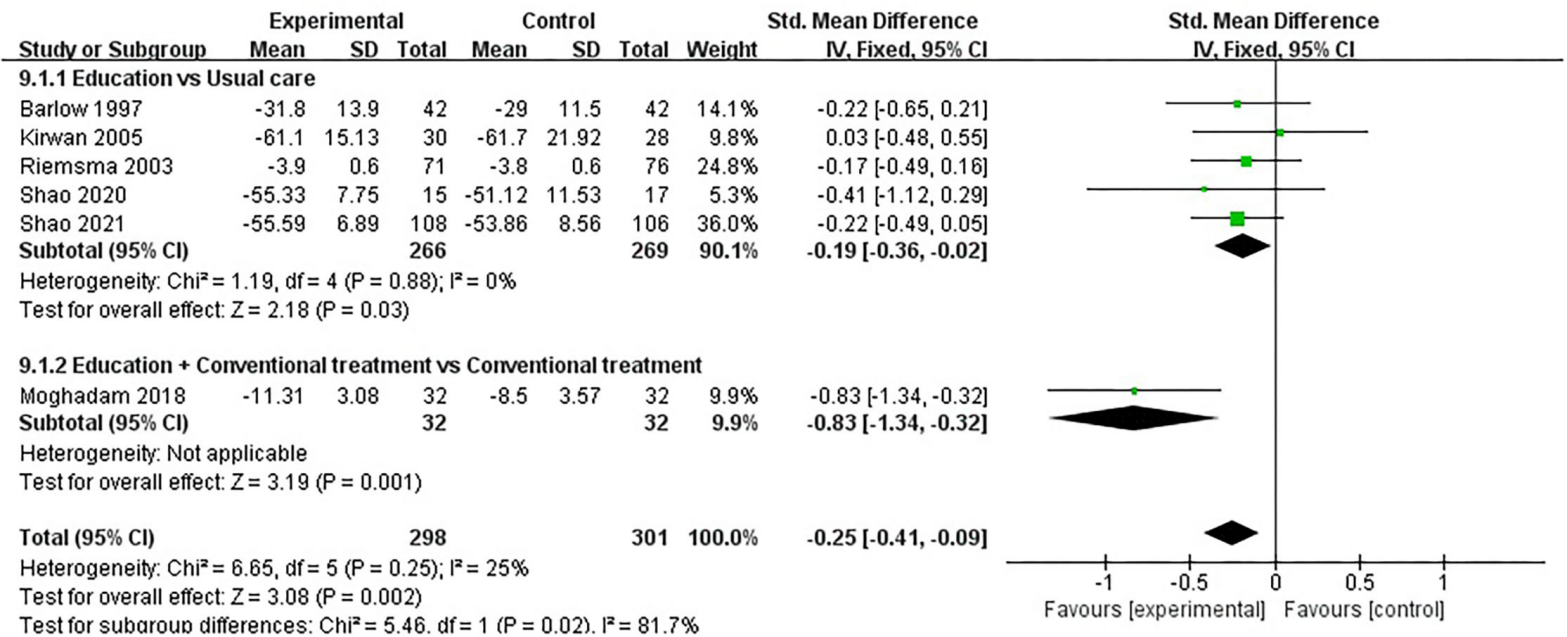
Meta-analysis on ASE (other symptoms).

ASE (other symptoms) was evaluated in six studies involving a total of 599 participants. Six studies assessed ASE (other symptoms) using the ASE scale (32, 35, 37, 49, 52, 54). Of the six RCTs, one studies reported that patient education improved ASE (other symptoms) in patients with RA (*P* < 0.05) (49), while the other five studies reported that patient education did not improve ASE (other symptoms) in patients with RA (*P* > 0.05) (32, 35, 37, 52, 54). Six RCTs were included in the meta-analysis (32, 35, 37, 49, 52, 54). Pooled results showed a significant improvement in ASE (other symptoms) in the education group compared to the control group [SMD = −0.25, 95% CI (−0.41, −0.09), *I*^2^ = 25%, *P* = 0.002]. When education was compared with usual care, the subgroup analysis showed a significant improvement in ASE (other symptoms) in the education group [SMD = −0.19, 95% CI (−0.36, −0.02), *I*^2^ = 0%, *P* = 0.03]. Similarly, when education + conventional treatment was compared with conventional treatment alone, subgroup analysis showed a significant improvement in ASE (other symptoms) [SMD = −0.83, 95% CI (−1.34, −0.32), *P* = 0.001] (Figure 10).

#### ASE (Total)

**Figure 11:**
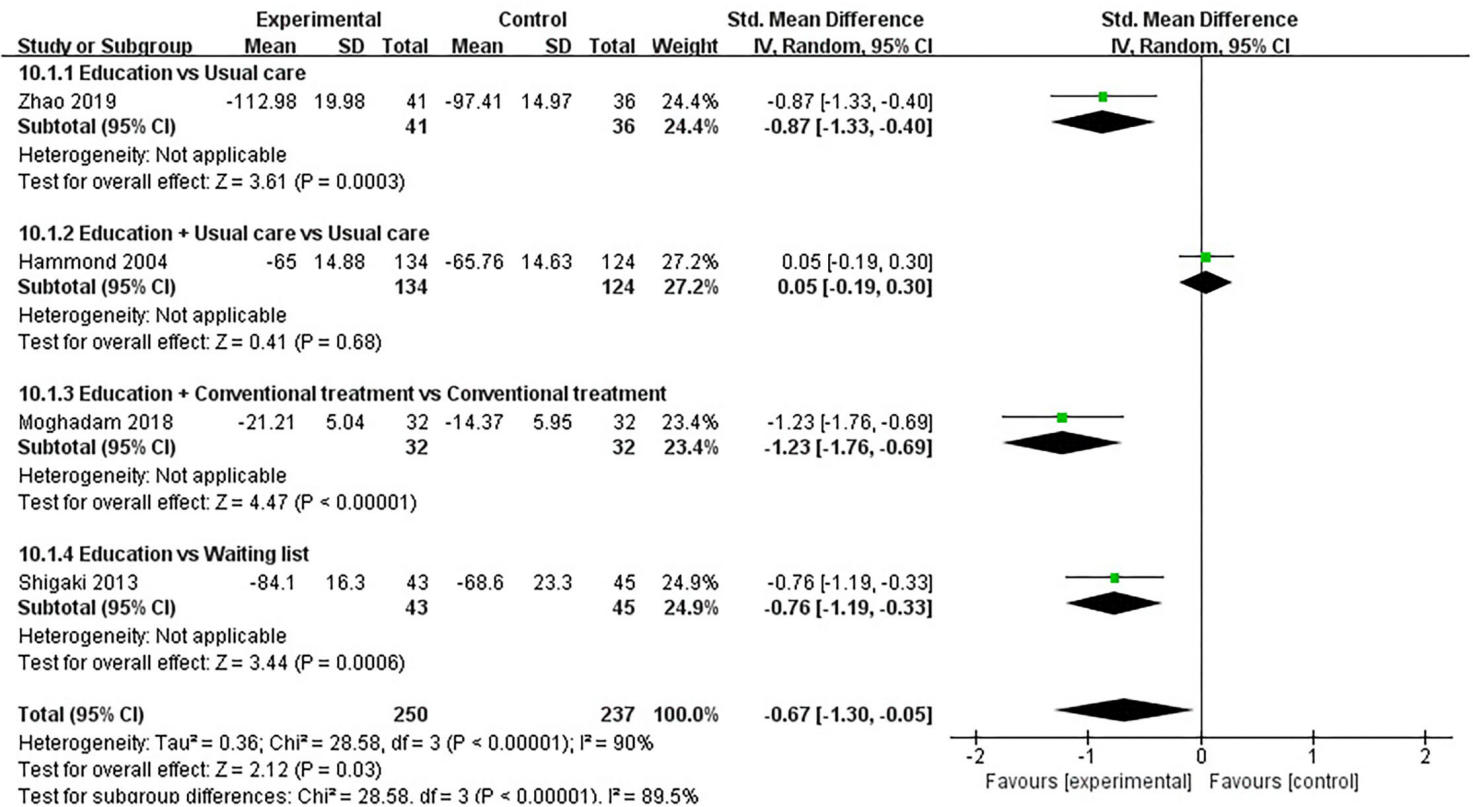
Meta-analysis on ASE (total).

ASE (total) was evaluated in four studies involving a total of 487 participants. Four studies assessed ASE (total) using the ASE scale (36, 45, 49, 51). Of the four RCTs, three studies reported that patient education improved ASE (total) in patients with RA (*P* < 0.05) (45, 49, 51), while the other study reported that patient education did not improve ASE (total) in patients with RA (*P* > 0.05) (36). Four RCTs were included in the meta-analysis (36, 45, 49, 51). Pooled results showed a significant improvement in ASE (total) in the education group compared to the control group [SMD = −0.67, 95% CI (−1.30, −0.05), *I*^2^ = 90%, *P* = 0.03]. When education was compared with usual care, the subgroup analysis showed a significant improvement in ASE (total) in the education group [SMD = −0.87, 95% CI (−1.33, −0.40), *P* = 0.0003]. When education + conventional treatment was compared with conventional treatment alone, the subgroup analysis showed a significant improvement in ASE (total) in the education group [SMD = −1.23, 95% CI (−1.76, −0.69), *P* < 0.00001]. Similarly, When education was compared waiting list (no intervention), the subgroup analysis showed a significant improvement in ASE (total) in the education group [SMD = −0.76, 95% CI (−1.19, −0.33), *P* = 0.0006]. However, when education + usual care was compared with usual care alone, the subgroup analysis revealed that the education group showed no significant difference in improved ASE (total) [SMD = −0.05, 95% CI (−0.30, 0.19), *P* = 0.68] (Figure 11).

#### General Health

**Figure 12:**
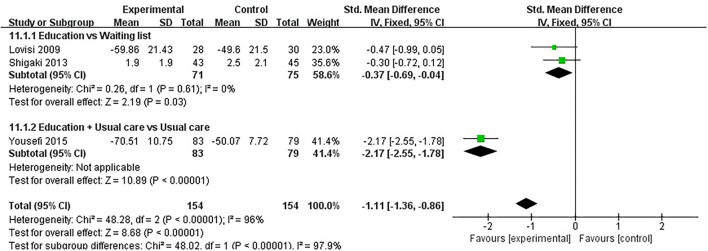
Meta-analysis on General health.

General health was evaluated in four studies involving a total of 340 participants. Four studies assessed general health using SF-36 (41, 46), EQ5D (42), or AIMS2 (45), respectively. The lower the AIMS2 score, the better the general health, and the higher the SF-36 and EQ5D, the better the general health. Of the four RCTs, two studies reported that patient education improved general health in patients with RA (*P* < 0.05) (42, 46), while the other two studies reported that patient education did not improve general health in patients with RA (*P* > 0.05) (41, 45). One RCT was not included in the meta-analysis (42). Three of the four RCTs were included in the meta-analysis (41, 45, 46). Pooled results showed a significant improvement in general health in the education group compared to the control group [SMD = −1.11, 95% CI (−1.36, −0.86), *I*^2^ = 96%, *P* < 0.00001]. When education was compared waiting list (no intervention), the subgroup analysis showed a significant improvement in general health in the education group [SMD = −0.37, 95% CI (−0.69, −0.04), *I*^2^ = 0%, *P* = 0.03]. Similarly, when education + usual care was compared with usual care alone, subgroup analysis showed a significant improvement in general health [SMD = −2.17, 95% CI (−2.55, −1.78), *P* < 0.00001] (Figure 12).

#### Publication Bias

When the meta-analysis includes more than ten studies, the possibility of publication bias should be reported (55). We used the Egger's and Begg's test to assess the publication bias of each outcome (30). The evaluation results showed that there was no publication bias in the outcomes (Table 4).

**Table 4:** Assessment of publication bias.

**Outcomes**	**No. of studies**	**Egger's test (*P*-values)**	**Begg's test (*P*-values)**
Pain	12	0.595^#^	0.451^#^
Physical function	15	0.278^#^	0.075^#^

*^#^P > 0.05*.

#### Meta-Regression Analysis and Sensitivity Analysis

In this study, we evaluated a total of 10 outcomes. The outcomes with high heterogeneity were pain, physical function, disease activity, anxiety, depression, ASE (pain), ASE (total), and general health. We used meta-regression analysis and sensitivity analysis to explore the sources of heterogeneity. Results of meta-regression analysis and sensitivity analysis were presented in Supplementary Appendix.

## Discussion

We conducted this review to evaluate evidence for the benefits of patient education interventions in people with RA. The outcomes include pain, physical function, disease activity, erythrocyte sedimentation rate (ESR), C-reactive protein (CRP), anxiety, depression, ASE (pain, other symptoms, total), and general health. The results of this meta-analysis suggest that patient education may be beneficial for improving physical function, disease activity, increasing ASE (pain), ASE (other symptoms), and ASE (total), and general health in patients with RA. However, there was no significant effect on anxiety, depression, pain, and CRP in patients with RA.

This study shows that patient education is generally beneficial for rheumatoid arthritis. Based on our previous work, we expected that patient education would contribute to clinical outcomes and psychological status in rheumatoid arthritis. Several previous studies have explored the impact of patient education on diseases such as low back pain and rheumatoid arthritis (26, 56). These studies found that patient education improved pain and general health in patients with acute low back pain, and improved general health, mental health, and depression in rheumatoid arthritis but did not significantly improve anxiety or disease activity. Our study found that patient education can help improve clinical outcomes in rheumatoid arthritis, such as physical function, disease activity, increasing ASE (pain), ASE (other symptoms), and ASE (total), and general health. The positive effect of patient education may be closely related to the active participation of patients, and it is very important for patients to adhere to rheumatoid arthritis-related interventions. However, our study found that patient education did not significantly improve anxiety, depression, pain, and CRP in patients with rheumatoid arthritis, and further research in this part is needed in the future. Overall, our results are in line with our prior expectations.

RA is a chronic inflammatory joint disease that can occur at any age, causing disability, loss of workability, and even death in patients with RA (57). Many patients with RA continue to experience joint pain, deformities, disability, and poor quality of life despite professional treatment (24). There are some interventions that can enhance the effectiveness of RA, and patient education is one of them, this may be related to patient participation and shared decision making. It can help patients understand RA and strengthen the management of the disease (58, 59). Patient education has been defined as “any set of planned educational activities designed to improve patients' health behaviors and health status” (24). EULAR recommends that patient education should be part of the treatment of RA (17). Studies have shown that patients may play an important role in the disease management of RA (39), but this important role may require active participation and shared decision making by patients, and patients must contribute actively to adhere to interventions for their RA. Health education for patients with RA can have a positive impact on the perception of pain and the management of the disease, and they transform the knowledge of the disease and the methods of preventing pain into changes in health behavior, which not only reduces the symptoms of pain and disability but also it will also improve body function (39, 60, 61). Antirheumatoid medications and biological agents can effectively control the symptoms and inflammatory response of RA, but the effectiveness of the medications is severely limited by patient adherence (8), and patient education can enhance the adherence of patients with RA and improve the effectiveness of medications (53), this may have a positive effect on improving their pain, physical function and reducing inflammatory cytokines (such as ESR, CRP, and RF). For patients with RA, self-efficacy is an important influencing factor to comply with health advice and health outcomes (62). The study found that the self-efficacy of patients with RA is significantly related to their health status (63, 64) and that patient education has a positive impact on clinical efficacy through self-efficacy (65). In addition, the effect of patient education on patients with RA was also affected by the education content. Some education content includes guiding patients to take family exercise, and family exercise has been proved to promote the maintenance of muscle strength, increase handgrip strength and flexibility (66–68), which can further improve the physical function of patients with RA. Clinical studies have shown that patient education was closely related to patients' mental health, especially in anxiety and depression, and patient education can improve patients' anxiety and depression (32, 69). The symptoms of RA are the result of the interaction between the body and the mind, and the symptoms of RA and psychological factors are closely related (69). Health education for patients with RA can increase the understanding of the disease and its treatment methods, which is beneficial to improve the anxiety and depression of patients with RA, and may also have a significant impact on the control of the disease. The study found that long-term behavior change of patients with RA through education can have a positive impact on disease control and also significantly reduce the disease activity of RA (19). Other studies have found that patient education may not have a direct impact on disease activity but maybe in an indirect way (17).

Patient education is increasing of interest to healthcare workers as a complementary intervention. Patient education has been recognized by many clinicians and is widely used in clinical practice. Some medical, orthopedic, and other diseases use patient education as an intervention, such as knee osteoarthritis, cancer, stroke, etc. (70–72). However, the mechanisms by which patient education works are complex and integrative. Some studies have found that patient education can reduce patient pain and function, and the mechanism by which patient education affects health outcomes may be related to self-efficacy (73). Patient education can improve patient self-efficacy, and some information, including exercise and health management, can promote healthy behavior changes (70, 73). Increased patient awareness of the disease can promote patient self-motivation, change their behavior, enhance disease management further, as well as increase patient adherence (74). In addition, patient education can improve psychosocial support, which contributes to disease management to a certain extent and promotes changes in patient behavior (75). Several studies suggest that patient education interventions should not be limited to treatment adherence alone but should also focus on the patient's mental health, which is valuable by combining psychosocial factors, clinical outcomes, and self-reported adherence (75, 76). Patient education is a long-term ongoing process and should be implemented with periodic assessment of its benefits and updating its interventions as necessary to accommodate changes in the patient's disease (76). The effectiveness of patient education may be affected by multiple factors, such as the patient's learning ability, literacy level, cultural environment, etc. Therefore, careful and comprehensive consideration should be given to developing interventions for patient education (77). In addition, some studies suggest that effective patient education should encourage patients to enhance self-efficacy and self-management, improve patient satisfaction, and effectively promote collaboration and communication between patients and professionals (38, 78–80).

### Limitations

This systematic review and meta-analysis had several limitations. Firstly, although all the included RCTs were randomized, it was not clear whether some included RCTs had a bias in allocation concealment and bias in performance bias (blinding of participants and personnel) and detection bias (blinding of outcome assessment). Therefore, the quality of evidence for outcomes had been reduced to low levels. Secondly, the sample size included in the study was small. Although a small sample size can be used for meta-analysis, the results might be biased, and the conclusions drawn should be considered preliminary (81). Thirdly, the RCTs varied in terms of their patient populations (such as disease duration), comparative treatments (specific methods, the length of intervention time, and frequency of interventions), and outcome measures. Therefore, the results of the meta-analysis showed that there was a high degree of heterogeneity. However, due to the small number of included RCTs and the fact that some RCTs did not specifically report these differences, we could not conduct a subgroup analysis to check whether these factors had an impact on the outcome of the study.

### Implications for Further Research and Practice

Considering that patient education is an intervention without side effects and helps to improve the curative effect of rheumatoid arthritis and control the development of rheumatoid arthritis, it is recommended that patient education be used as a treatment strategy for rheumatoid arthritis. The use of patient education as an intervention for RA is of great significance for reducing the medical cost of RA, especially in developing countries. In addition, this study also provides data for future clinical research on RA. However, due to the low level of evidence in this study and the high heterogeneity of the study, more high-quality RCTs should be conducted in the future to verify these conclusions. We acknowledge the difficulty of conducting RCTs in this study, but some methods can make future study designs more comprehensive and rigorous. Firstly, the implementation of RCTs on patient education in the future should strictly follow the Consolidated Standards of Reporting Trials (CONSORT) statement to improve the quality of research (82). Secondly, researchers should complete registration at a standard clinical study center before starting a clinical study, and complete study protocols should be published to reduce publication bias (83). Finally, clinical studies should extend the duration of follow-up and increase the frequency of follow-up to assess the long-term efficacy of interventions.

Patient education is a broad-based intervention that may serve as a foundational intervention for many diseases. However, patient education is effective on clinical outcomes remains inconclusive. Studies have found that it is difficult to compare the effects of patient education on different chronic diseases and also among different types of arthritis (84). Therefore, we are only studying rheumatoid arthritis and not simultaneously studying and comparing larger diseases, such as rheumatic diseases, osteoarthritis, or other chronic musculoskeletal diseases. In the future, our research should be extended to other arthritis or chronic musculoskeletal diseases to explore further the impact of patient education on other arthritic or chronic musculoskeletal diseases. At the same time, patient education can be considered as a disease-based intervention, combining patient education with other interventions.

## Conclusion

Patient education may be effective in improving clinical outcomes and psychological status in patients with rheumatoid arthritis. Considering the methodological limitations of the included RCTs, more high-quality and large-sample RCTs are needed to confirm this conclusion in the future.

## Data Availability Statement

The original contributions presented in the study are included in the article/Supplementary Material, further inquiries can be directed to the corresponding author/s.

## Author Contributions

ZGW, WGL, and XMX designed the study. ZGW, YZ, YW, and RZ conducted a literature search and screening and extracted data from the literature. XLY, ZHC, CCL, and JYL checked the extracted data. ZGW, ZBW, and ZXY conducted the statistical analysis. ZGW, YZ, YW, and RZ wrote the first draft. WGL and XMX corrected the manuscript. ZXY did the language editing. WGL and XMX supervised the conduct of the study. All authors have read and approved the final submitted version.

## Funding

This work was supported by Guangdong Provincial Science and Technology Innovation Strategy Special Fund (2021B1111610007) and Natural Science Foundation of Guangdong Province (2021A1515011545).

## Conflict of Interest

The authors declare that the research was conducted in the absence of any commercial or financial relationships that could be construed as a potential conflict of interest.

## Publisher's Note

All claims expressed in this article are solely those of the authors and do not necessarily represent those of their affiliated organizations, or those of the publisher, the editors and the reviewers. Any product that may be evaluated in this article, or claim that may be made by its manufacturer, is not guaranteed or endorsed by the publisher.

## References

[1] Sparks Jeffrey A. Rheumatoid arthritis. Ann Intern Med. (2019) 170: ITC1–16. 10.7326/AITC201901010 30596879

[2] Smolen JS Aletaha D Barton A Burmester GR Emery P Firestein GS . Rheumatoid arthritis. Nat Rev Dis Primers. (2018) 4:18001. 10.1038/nrdp.2018.1 29417936

[3] Kitas GD Gabriel S E. Cardiovascular disease in rheumatoid arthritis: state of the art and future perspectives. Ann Rheum Dis. (2011) 70:8–14. 10.1136/ard.2010.142133 21109513

[4] Sokka T Kautiainen H Pincus T Verstappen SMM Aggarwal A Alten R . Work disability remains a major problem in rheumatoid arthritis in the 2000s: data from 32 countries in the QUEST-RA study. Arthritis Res Ther. (2010) 12:R42. 10.1186/ar2951 20226018 PMC2888189

[5] Cross M Smith E Hoy D Carmona L Wolfe F Vos T . The global burden of rheumatoid arthritis: estimates from the global burden of disease 2010 study. Ann Rheum Dis. (2014) 73:1316–22. 10.1136/annrheumdis-2013-204627 24550173

[6] Myasoedova E Davis J Matteson EL Crowson C S. Is the epidemiology of rheumatoid arthritis changing? Results from a population-based incidence study, 1985-2014. Ann Rheum Dis. (2020) 79:440–4. 10.1136/annrheumdis-2019-216694 32066556 PMC7085464

[7] Smolen JS Landewé R Bijlsma J Burmester G Chatzidionysiou K Dougados M . EULAR recommendations for the management of rheumatoid arthritis with synthetic and biological disease-modifying antirheumatic drugs: 2016 update. Ann Rheum Dis. (2017) 76:960–77. 10.1136/annrheumdis-2016-210715 28264816

[8] Scheiman-Elazary A Duan L Shourt C Agrawal H Ellashof D Cameron-Hay M . The rate of adherence to antiarthritis medications and associated factors among patients with rheumatoid arthritis: a systematic literature review and meta-analysis. J Rheumatol. (2016) 43:512–23. 10.3899/jrheum.141371 26879354

[9] Dolati S Sadreddini S Rostamzadeh D Ahmadi M Jadidi-Niaragh F Yousefi M. Utilization of nanoparticle technology in rheumatoid arthritis treatment. Biomed Pharmacother. (2016) 80:30–41. 10.1016/j.biopha.2016.03.004 27133037

[10] Cohen MJ Shaykevich S Cawthon C Kripalani S Paasche-Orlow MK Schnipper J L. Predictors of medication adherence postdischarge: the impact of patient age, insurance status, and prior adherence. J Hosp Med. (2012) 7:470–5. 10.1002/jhm.1940 22473754 PMC3575732

[11] van den Bemt BJ Zwikker HE van den Ende CH. Medication adherence in patients with rheumatoid arthritis: a critical appraisal of the existing literature. Expert Rev Clin Immunol. (2012) 8:337–51. 10.1586/eci.12.23 22607180

[12] Achaval SD Suarez-Almazor ME. Treatment adherence to disease-modifying antirheumatic drugs in patients with rheumatoid arthritis and systemic lupus erythematosus. Int J Clin Rheumtol. (2010) 5:313–26. 10.2217/ijr.10.15 20676388 PMC2910438

[13] Ritschl V Stamm TA Aletaha D Bijlsma JWJ Böhm P Dragoi RG . 2020 EULAR points to consider for the prevention, screening, assessment and management of non-adherence to treatment in people with rheumatic and musculoskeletal diseases for use in clinical practice. Ann Rheum Dis. (2020) 18:707–13. 10.1136/annrheumdis-2020-218986 33355152

[14] Birgitta N Cecilia F Ingrid D Gunnar B Lundberg IE Dufour AB . An outsourced health-enhancing physical activity programme for people with rheumatoid arthritis: exploration of adherence and response. Rheumatology. (2015) 6:1065–073. 10.1093/rheumatology/keu444 25433043 PMC4481374

[15] Bech B Primdahl J van Tubergen A Voshaar M Zangi HA Barbosa L . 2018 update of the EULAR recommendations for the role of the nurse in the management of chronic inflammatory arthritis. Ann Rheum Dis. (2020) 79:61–8. 10.1136/annrheumdis-2019-215458 31300458

[16] M'imunya JM Kredo T Volmink J. Patient education and counselling for promoting adherence to treatment for tuberculosis. Cochrane Database Syst Rev. (2012) 5:CD006591. 10.1002/14651858.CD006591.pub2 22592714 PMC6532681

[17] Zangi HA Ndosi M Adams J Andersen L Bode C Boström C . European League Against Rheumatism (EULAR). EULAR recommendations for patient education for people with inflammatory arthritis. Ann Rheum Dis. (2015) 74:954–62. 10.1136/annrheumdis-2014-206807 25735643

[18] Aliha JM Asgari M Khayeri F Ramazani M Farajzadegan Z Javaheri J. Group education and nurse-telephone follow-up effects on blood glucose control and adherence to treatment in type 2 diabetes patients. Int J Prev Med. (2013) 4:797–802. 24049598 PMC3775219

[19] Miedany YE Gaafary ME Arousy NE Ahmed I Youssef S Palmer D. Arthritis education: the integration of patient-reported outcome measures and patient self-management. Clin Exp Rheumatol. (2012) 30:899–904. 22992291

[20] Ravindran V Jadhav R. The effect of rheumatoid arthritis disease education on adherence to medications and followup in Kerala, India. J Rheumatol. (2013) 40:1460–1. 10.3899/jrheum.130350 23908549

[21] Lorig KR Mazonson PD Holman H R. Evidence suggesting that health education for self-management in patients with chronic arthritis has sustained health benefits while reducing health care costs. Arthritis Rheum. (1993) 36:439–46. 10.1002/art.1780360403 8457219

[22] Galo JS Mehat P Rai SK Avina-Zubieta A Vera M A D. What are the effects of medication adherence interventions in rheumatic diseases: a systematic review. Ann Rheum Dis. (2016) 75:667–73. 10.1136/annrheumdis-2014-206593 25667208

[23] Shadick NA Zibit MJ Iannaccone CK Thrower R Sowell NF Weinblatt ME . A development and feasibility study of a peer support telephone program in rheumatoid arthritis. J Clin Rheumatol. (2018) 24:346–9. 10.1097/RHU.0000000000000661 29389689 PMC6699178

[24] Riemsma RP Taal E Kirwan JR Rasker J J. Systematic review of rheumatoid arthritis patient education. Arthritis Rheum. (2004) 51:1045–59. 10.1002/art.20823 15593105

[25] Carandang K Pyatak EA Vigen CL. Systematic review of educational interventions for rheumatoid arthritis. Am J Occup Ther. (2016) 70:7006290020p1–12. 10.5014/ajot.2016.021386 27767950

[26] Riemsma RP Kirwan JR Taal E Rasker JJ. Patient education for adults with rheumatoid arthritis. Cochrane Database Syst Rev. (2003) CD003688. 10.1002/14651858.CD003688 12804484

[27] Shamseer L Moher D Clarke M Ghersi D Liberati A Petticrew M . PRISMA-P Group. Preferred reporting items for systematic review and meta-analysis protocols (PRISMA-P) 2015: elaboration and explanation. BMJ. (2015) 350:g7647. 10.1136/bmj.g7647 25555855

[28] Lorig K. Common Sense Patient Education. Ivanhoe, VIC: Fraser Publications (1992).

[29] Tarsilla M. Cochrane handbook for systematic reviews of interventions. J Multidisc Eval. (2010) 6:142–8.31643080

[30] Egger M Davey Smith G Schneider M Minder C. Bias in meta-analysis detected by a simple, graphical test. BMJ. (1997) 315:629–34. 10.1136/bmj.315.7109.629 9310563 PMC2127453

[31] Helewa A Goldsmith CH Lee P Bombardier C Hanes B Smythe HA . Effects of occupational therapy home service on patients with rheumatoid arthritis. Lancet. (1991) 337:1453–6. 10.1016/0140-6736(91)93138-Y 1675329

[32] Barlow JH Wright CC. Knowledge in patients with rheumatoid arthritis: a longer term follow-up of a randomized controlled study of patient education leaflets. Br J Rheumatol. (1998) 37:373–6. 10.1093/rheumatology/37.4.373 9619885

[33] Scholten C Brodowicz T Graninger W Gardavsky I Pils K Pesau B . Persistent functional and social benefit 5 years after a multidisciplinary arthritis training program. Arch Phys Med Rehabil. (1999) 80:1282–7. 10.1016/S0003-9993(99)90030-8 10527088

[34] Hill J Bird H Johnson S. Effect of patient education on adherence to drug treatment for rheumatoid arthritis: a randomised controlled trial. Ann Rheum Dis. (2001) 60:869–75. 11502614 PMC1753835

[35] Riemsma RP Taal E Rasker J J. Group education for patients with rheumatoid arthritis and their partners. Arthritis Rheum. (2003) 49:556–66. 10.1002/art.11207 12910564

[36] Hammond A Young A Kidao R. A randomised controlled trial of occupational therapy for people with early rheumatoid arthritis. Ann Rheum Dis. (2004) 63:23–30. 10.1136/ard.2002.001511 14672887 PMC1754722

[37] Kirwan JR Hewlett S Cockshott Z Barrett J. Clinical and psychological outcomes of patient education in rheumatoid arthritis. Musculoskel Care. (2005) 3:1–16. 10.1002/msc.21 17041989

[38] Núñez M Núñez E Yoldi C Quintó L Hernández MV Muñoz-Gómez J. Health-related quality of life in rheumatoid arthritis: therapeutic education plus pharmacological treatment versus pharmacological treatment only. Rheumatol Int. (2006) 26:752–7. 10.1007/s00296-005-0071-6 16247548

[39] Masiero S Boniolo A Wassermann L Machiedo H Volante D Punzi L. Effects of an educational-behavioral joint protection program on people with moderate to severe rheumatoid arthritis: a randomized controlled trial. Clin Rheumatol. (2007) 26:2043–50. 10.1007/s10067-007-0615-0 17404783

[40] Giraudet-Le Quintrec JS Mayoux-Benhamou A Ravaud P Champion K Dernis E Zerkak D . Effect of a collective educational program for patients with rheumatoid arthritis: a prospective 12-month randomized controlled trial. J Rheumatol. (2007) 34:1684–91. 17610321

[41] Lovisi Neto BE Jennings F Barros Ohashi C Silva PG Natour J. Evaluation of the efficacy of an educational program for rheumatoid arthritis patients. Clin Exp Rheumatol. (2009) 27:28–34. 19327226

[42] Macedo AM Oakley SP Panayi GS Kirkham B W. Functional and work outcomes improve in patients with rheumatoid arthritis who receive targeted, comprehensive occupational therapy. Arthritis Rheum. (2009) 61:1522–30. 10.1002/art.24563 19877106

[43] Mathieux R Marotte H Battistini L Sarrazin A Berthier M Miossec P. Early occupational therapy programme increases hand grip strength at 3 months: results from a randomised, blind, controlled study in early rheumatoid arthritis. Ann Rheum Dis. (2009) 68:400–3. 10.1136/ard.2008.094532 19015209

[44] Conn DL Pan Y Easley KA Comeau DL Carlone JP Culler SD . The effect of the arthritis self-management program on outcome in african americans with rheumatoid arthritis served by a public hospital. Clin Rheumatol. (2013) 32:49–59. 10.1007/s10067-012-2090-5 23053684

[45] Shigaki CL Smarr KL Siva C Ge B Musser D Johnson R. RAHelp: an online intervention for individuals with rheumatoid arthritis. Arthritis Care Res. (2013) 65:1573–81. 10.1002/acr.22042 23666599

[46] Yousefi H Chopra A Farrokhseresht R Sarmukaddam S Noghabi FA Bedekar N . Epidemiological evaluation quality of life in patients suffering from early rheumatoid arthritis: a pragmatic, prospective, randomized, blind allocation controlled of a modular program group intervention. Epidemiol Health. (2015) 37:e2015048. 10.4178/epih/e2015048 26552423 PMC4860498

[47] Pot-Vaucel M Aubert MP Guillot P Glémarec J Berthelot JM Le Goff B . Randomised study versus control group of customised therapeutic education for patients in follow-up for rheumatoid arthritis. Joint Bone Spine. (2016) 83:199–6. 10.1016/j.jbspin.2015.05.017 26677992

[48] Anvar N Matlabi H Safaiyan A Allahverdipour H Kolahi S. Effectiveness of self-management program on arthritis symptoms among older women: a randomized controlled trial study. Health Care Women Int. (2018) 39:1326–39. 10.1080/07399332.2018.1438438 29419362

[49] Hosseini Moghadam M Jahanbin I Nazarinia M A. The effect of educational program on self-efficacy of women with rheumatoid arthritis: a randomized controlled clinical trial. Int J Community Based Nurs Midwifery. (2018) 6:12–20. 29399585 PMC5747567

[50] Saeedifar ES Memarian R Fatahi S Ghelichkhani F. Use of the Orem self-care model on pain relief in women with rheumatoid arthritis: a randomized trial. Electron Phys. (2018) 10:6884–91. 10.19082/6884 30034655 PMC6049968

[51] Zhao SP Chen H. Effectiveness of health education by telephone follow-up on self-efficacy among discharged patients with rheumatoid arthritis: a randomised control trial. J Clin Nurs. (2019) 28:3840–7. 10.1111/jocn.15002 31325348

[52] Shao J H., Yu K H., Chen S H. Feasibility and acceptability of a self-management program for patients with rheumatoid arthritis. Orthop Nurs. (2020) 39:238–45. 10.1097/NOR.0000000000000676 32701780

[53] Song Y Reifsnider E Zhao S Xie X Chen H. A randomized controlled trial of the Effects of a telehealth educational intervention on medication adherence and disease activity in rheumatoid arthritis patients. J Adv Nurs. (2020) 76:1172–81. 10.1111/jan.14319 32026506

[54] Shao JH Yu KH Chen S H. Effectiveness of a self-management program for joint protection and physical activity in patients with rheumatoid arthritis: a randomized controlled trial. Int J Nurs Stud. (2021) 116:103752. 10.1016/j.ijnurstu.2020.103752 32928503

[55] Sterne JAC Sutton AJ Ioannidis JPA Terrin Norma Jones David R Lau Joseph . Recommendations for examining and interpreting funnel plot asymmetry in meta-analyses of randomised controlled trials. BMJ. (2011) 343:d4002. 10.1136/bmj.d4002 21784880

[56] Engers A Jellema P Wensing M van der Windt DA Grol R van Tulder MW. Individual patient education for low back pain. Cochrane Database Syst Rev. (2008) 2008:CD004057. 10.1002/14651858.CD004057.pub3 18254037 PMC6999124

[57] Aletaha D Smolen J S. Diagnosis and management of rheumatoid arthritis: a review. JAMA. (2018) 320:1360–72. 10.1001/jama.2018.13103 30285183

[58] Kirwan J R. Patient education in rheumatoid arthritis. Curr Opin Rheumatol. (1990) 2:336–9. 10.1097/00002281-199002020-00015 2203411

[59] Taal E Rasker JJ Wiegman O. Patient education and self-management in the rheumatic diseases: a self-efficacy approach. Arthritis Care Res. (1996) 9:229–38. 8971233 10.1002/1529-0131(199606)9:3<229::aid-anr1790090312>3.0.co;2-u

[60] Neuberger GB Smith KV Black SO Hassanein R. Promoting self-care in clients with arthritis. Arthritis Care Res. (1993) 6:141–8. 10.1002/art.1790060306 8130290

[61] Taal E Rasker JJ Wiegman O. Group education for rheumatoid arthritis patients. Semin Arthritis Rheumat. (1997) 26:805–16. 10.1016/S0049-0172(97)80024-8 9213379

[62] Taal E Seydel ER Rasker JJ Wiegman O. Psychosocial aspects of rheumatic diseases: introduction. Pat Educ Counsel. (1993) 20:55–61. 10.1016/0738-3991(93)90121-C 8337195

[63] Keefe FJ Van Horn Y. Cognitive-behavioral treatment of rheumatoid arthritis pain: maintaining treatment gains. Arthritis Care Res. (1993) 6:213–22. 10.1002/art.1790060408 7918717

[64] Schiaffino KM Revenson TA Gibofsky A. Assessing the impact of self-efficacy beliefs on adaptation to rheumatoid arthritis. Arthritis Care Res. (1991) 4:150–7. 10.1002/art.1790040404 11188602

[65] Lorig K Seleznick M Lubeck D Ung E Chastain R L., Holman H R. The beneficial outcomes of the arthritis self-management course are not adequately explained by behavior change. Arthritis Rheum. (1989) 32:91–5. 10.1002/anr.1780320116 2912467

[66] Mccubbin J A. Resistance exercise training for persons with arthritis. Rheum Dis Clin N Am. (1990) 16:931–43. 10.1016/S0889-857X(21)00916-9 2087585

[67] Brighton SW Lubbe JE van der Merwe C A. The effect of a long-term exercise programme on the rheumatoid hand. Br J Rheumatol. (1993) 32:392–5. 10.1093/rheumatology/32.5.392 8495260

[68] Hoenig H Groff G Pratt K Goldberg E Franck W. A randomized controlled trial of home exercise on the rheumatoid hand. J Rheumatol. (1993) 20:785–9. 8336303

[69] Barsky AJ Ahern DK Orav EJ Nestoriuc Y Liang Ma H Berman IT . A randomized trial of three psychosocial treatments for the symptoms of rheumatoid arthritis. Semin Arthritis Rheum. (2010) 40:222–32. 10.1016/j.semarthrit.2010.04.001 20621334 PMC2993818

[70] Taglietti M Facci LM Trelha CS de Melo FC da Silva DW Sawczuk G . Effectiveness of aquatic exercises compared to patient-education on health status in individuals with knee osteoarthritis: a randomized controlled trial. Clin Rehabil. (2018) 32:766–76. 10.1177/0269215517754240 29417831

[71] Reif K de Vries U Petermann F Görres S. A patient education program is effective in reducing cancer-related fatigue: a multi-centre randomised two-group waiting-list controlled intervention trial. Eur J Oncol Nurs. (2013) 17:204–13. 10.1016/j.ejon.2012.07.002 22898654

[72] Chu YM Choi KS. Effectiveness of patient education in acute stroke: a comparison between a customised computer system and a pictorial information booklet. BMJ Health Care Inform. (2020) 27:e100144. 10.1136/bmjhci-2020-100144 32816839 PMC7430415

[73] Pajares F. Overview of Social Cognitive Theory and Self-Efficacy Atlanta: Division of Educational Studies. Atlanta, GA: Emory College of Arts and Sciences, Emory University (2002).

[74] Elder J Ayala G Harris S. Theories and intervention approaches to health-behaviour change in primary care. Am J Prev Med. (1999) 4:275–84. 10.1016/S0749-3797(99)00094-X 10606196

[75] Welch JL Thomas-Hawkins C. Psycho-educational strategies to promote fluid adherence in adult hemodialysis patients: a review of intervention studies. Int J Nurs Stud. (2005) 42:597–608. 10.1016/j.ijnurstu.2004.09.015 15921991

[76] Idier L Untas A Koleck M Chauveau P Rascle N. Assessment and effects of Therapeutic Patient Education for patients in hemodialysis: a systematic review. Int J Nurs Stud. (2011) 48:1570–86. 10.1016/j.ijnurstu.2011.08.006 21924423

[77] Marcus C. Strategies for improving the quality of verbal patient and family education: a review of the literature and creation of the EDUCATE model. Health Psychol Behav Med. (2014) 2:482–95. 10.1080/21642850.2014.900450 25750796 PMC4346059

[78] Schrieber L Colley M. Patient education. Best Pract Res Clin Rheumatol. (2004) 18:465–76. 10.1016/j.berh.2004.03.008 15301981

[79] Ndosi M Johnson D Young T Hardware B Hill J Hale C . Effects of needs-based patient education on self-efficacy and health outcomes in people with rheumatoid arthritis: a multicentre, single blind, randomised controlled trial. Ann Rheum Dis. (2016) 75:1126–32. 10.1136/annrheumdis-2014-207171 26162769 PMC4893097

[80] Hoving C Visser A Mullen PD van den Borne B. A history of patient education by health professionals in Europe and North America: from authority to shared decision making education. Patient Educ Couns. (2010) 78:275–81. 10.1016/j.pec.2010.01.015 20189746

[81] Higgins JPT Thomas J Chandler J Cumpston M Li T Page MJ . Cochrane Handbook for Systematic Reviews of Interventions. 2nd ed. Chichester: John Wiley & Sons (2019).

[82] Boutron I Moher D Altman D Schulz K Ravaud P CONSORT Group. Extending the CONSORT statement to randomized trials of nonpharmacologic treatment: explanation and elaboration. ACP J Club. (2008) 148:295–309. 10.7326/0003-4819-148-4-200802190-00008 18283207

[83] Wu Z Wang Y Ye X Chen Z Zhou R Ye Z . Myofascial release for chronic low back pain: a systematic review and meta-analysis. Front Med. (2021) 8:697986. 10.3389/fmed.2021.697986 34395477 PMC8355621

[84] Warsi A Wang PS LaValley MP Avorn J Solomon DH. Self-management education programs in chronic disease: a systematic review and methodological critique of the literature. Arch Intern Med. (2004) 164:1641–9. 10.1001/archinte.164.15.1641 15302634

