# Supplementary material for: The Effects of Patient Education on Psychological Status and Clinical Outcomes in Rheumatoid Arthritis: A Systematic Review and Meta-Analysis
Source: Front Psychiatry. 2022 Mar 17;13:848427. doi: 10.3389/fpsyt.2022.848427 (PMC8968629; doi:10.3389/fpsyt.2022.848427)
Supplement: Supplementary file 1 [file Data_Sheet_1.docx]

**TABLE S1 Meta-regression analysis.**

| **Variables** | **Regression coefficient (SE)** | **95% CI** | ***P* Value** |
| --- | --- | --- | --- |
| Pain (n=12) |  |  |  |
| Age | 0.060(0.027) | -0.007 to 0.127 | 0.072 |
| Sample size | -0.001(0.003) | -0.009 to 0.007 | 0.770 |
| Duration of intervention | -0.016(0.028) | -0.085 to 0.051 | 0.568 |
| Follow-up time | -0.000(0.008) | -0.020 to 0.019 | 0.924 |
| Physical function (n=15) |  |  |  |
| Age | 0.116(0.041) | 0.022 to 0.211 | 0.021^#^ |
| Sample size | 0.001(0.003) | -0.006 to 0.008 | 0.713 |
| Duration of intervention | 0.013(0.011) | -0.012 to 0.039 | 0.257 |
| Follow-up time | 0.001(0.009) | -0.020 to 0.023 | 0.885 |
| Disease activity (n=8) |  |  |  |
| Age | 1.649(0.946) | -1.362 to 4.661 | 0.180 |
| Sample size | 0.015(0.044) | -0.125 to 0.156 | 0.784 |
| Duration of intervention | -0.800(1.012) | -4.023 to 2.422 | 0.487 |
| Follow-up time | -0.527(0.318) | -1.539 to 0.485 | 0.196 |
| Depression (n=8) |  |  |  |
| Age | 0.033(0.034) | -0.015 to 0.182 | 0.437 |
| Sample size | -0.016(0.012) | -0.068 to 0.036 | 0.312 |
| Duration of intervention | 0.038(0.015) | -0.029 to 0.106 | 0.135 |
| Follow-up time | -0.002(0.012) | -0.056 to 0.050 | 0.841 |
| ASE(pain) (n=7) |  |  |  |
| Age | 0.302(0.136) | -0.047 to 0.652 | 0.077 |
| Sample size | -0.003(0.018) | -0.050 to 0.043 | 0.853 |
| Duration of intervention | -0.024(0.638) | -1.664 to 1.616 | 0.971 |
| Follow-up time | -0.062(0.063) | -0.225 to 0.101 | 0.373 |
| ASE(total) (n=4) |  |  |  |
| Age | -0.102(0.079) | -0.444 to 0.239 | 0.327 |
| Sample size | -0.005(0.001) | -0.009 to -0.001 | 0.034^#^ |
| Duration of intervention | 0.103(0.103) | -0.342 to 0.550 | 0.422 |
| Follow-up time | -0.014(0.002) | -0.025 to -0.002 | 0.033^#^ |
| General health (n=4) |  |  |  |
| Age | -0.294(0.259) | -3.595 to 3.006 | 0.460 |
| Sample size | 0.018(0.006) | -0.070 to 0.106 | 0.232 |
| Follow-up time | 0.052(0.022) | -0.239 to 0.344 | 0.264 |

^#^ indicates *P* < 0.05.

**Table S2 Sensitivity analysis for pain.**

| **Studies** | **Effect size** | **95% CI** | ***P* Value** | ***I* ^2^ %** |
| --- | --- | --- | --- | --- |
| Barlow (21) | -0.42 | -0.90 to 0.07 | 0.09 | 90 |
| Kirwan (26) | -0.46 | -0.91 to 0.01 | 0.05 | 90 |
| Hammond (25) | -0.43 | -0.92 to 0.07 | 0.09 | 89 |
| Yousefi (35) | -0.19 | -0.41 to 0.04 | 0.11 | 53 |
| Hill (23) | -0.37 | -0.86 to 0.12 | 0.14 | 91 |
| Masiero (28) | -0.39 | -0.87 to 0.09 | 0.11 | 91 |
| Saeedifar (39) | -0.31 | -0.77 to 0.15 | 0.19 | 90 |
| Shigaki (34) | -0.42 | -0.90 to 0.06 | 0.09 | 90 |

CI, confidence interval.

**Table S3 Sensitivity analysis for physical function.**

| **Studies** | **Effect size** | **95% CI** | ***P* Value** | ***I* ^2^ %** |
| --- | --- | --- | --- | --- |
| Conn (22) | -0.59 | -1.07 to -0.11 | 0.02 | 93 |
| Shao (43) | -0.52 | -1.03 to -0.01 | 0.05 | 93 |
| Zhao (40) | -0.56 | -1.05 to -0.08 | 0.02 | 93 |
| Hammond (25) | -0.58 | -1.08 to -0.07 | 0.03 | 93 |
| Quintrec (29) | -0.61 | -1.07 t0 -0.15 | 0.01 | 92 |
| Yousefi (35) | -0.30 | -0.56 to -0.04 | 0.02 | 75 |
| Masiero (28) | -0.51 | -0.99 to -0.03 | 0.04 | 93 |
| Montserrat (27) | -0.50 | -0.97 to -0.03 | 0.04 | 93 |
| Mathieux (32) | -0.51 | -0.99 to -0.03 | 0.04 | 93 |
| Scholten (22) | -0.49 | -0.97 to -0.02 | 0.04 | 93 |

CI, confidence interval.

**Table S4 Sensitivity analysis for disease activity.**

| **Studies** | **Effect size** | **95% CI** | ***P* Value** | ***I* ^2^ %** |
| --- | --- | --- | --- | --- |
| Shao (41) | -2.57 | -4.11 to -1.02 | 0.001 | 98 |
| Shao (43) | -2.87 | -4.87 to -0.87 | 0.005 | 97 |
| Zhao (40) | -2.66 | -4.42 to -0.90 | 0.003 | 98 |
| Song (42) | -2.64 | -4.40 to -0.89 | 0.003 | 98 |
| Masiero (28) | -0.31 | -0.50 to -0.11 | 0.002 | 0 |

CI, confidence interval.

**Table S5 Sensitivity analysis for depression.**

| **Studies** | **Effect size** | **95% CI** | ***P* Value** | ***I* ^2^ %** |
| --- | --- | --- | --- | --- |
| Barlow (21) | -0.11 | -0.56 to 0.35 | 0.64 | 63 |
| Marianne (36) | -0.34 | -0.59 to -0.08 | 0.01 | 0 |
| Scholten (22) | -0.10 | -0.52 to 0.33 | 0.65 | 61 |
| Shigaki (34) | -0.15 | -0.64 to 0.34 | 0.55 | 68 |

CI, confidence interval.

**Table S6 Sensitivity analysis for ASE(pain).**

| **Studies** | **Effect size** | **95% CI** | ***P* Value** | ***I* ^2^ %** |
| --- | --- | --- | --- | --- |
| Anvar (37) | -0.48 | -0.85 to -0.12 | 0.01 | 77 |
| Barlow (21) | -1.43 | -2.40 to -0.47 | 0.004 | 96 |
| Kirwan (26) | -1.43 | -2.37 to -0.49 | 0.003 | 96 |
| Riemsma (24) | -1.48 | -2.47 to -0.48 | 0.004 | 96 |
| Shao (41) | -1.45 | -2.35 to -0.55 | 0.002 | 96 |
| Shao (43) | -1.36 | -2.42 to -0.30 | 0.01 | 96 |
| Moghadam (38) | -1.27 | -2.20 to -0.34 | 0.007 | 96 |

CI, confidence interval; ASE, Arthritis Self-Efficacy Questionnaire.

**Table S7 Sensitivity analysis for ASE(total).**

| **Studies** | **Effect size** | **95% CI** | ***P* Value** | ***I* ^2^ %** |
| --- | --- | --- | --- | --- |
| Zhao (40) | -0.62 | -1.40 to 0.17 | 0.12 | 92 |
| Hammond (25) | -0.92 | -1.19 to -0.64 | 0.0001 | 0 |
| Moghadam (38) | -0.50 | -1.15 to 0.15 | 0.13 | 89 |
| Shigaki (34) | -0.65 | -1.49 to 0.18 | 0.12 | 92 |

CI, confidence interval; ASE, Arthritis Self-Efficacy Questionnaire.

**Table S8 Sensitivity analysis for General health.**

| **Studies** | **Effect size** | **95% CI** | ***P* Value** | ***I* ^2^ %** |
| --- | --- | --- | --- | --- |
| Lovisi (30) | -1.30 | -1.59 to -1.02 | <0.00001 | 98 |
| Shigaki (34) | -1.56 | -1.87 to -1.25 | <0.00001 | 96 |
| Yousefi (35) | -0.37 | -0.69 to -0.04 | 0.03 | 0 |

CI, confidence interval.
