# Supplementary material for: The Effects of Patient Education on Psychological Status and Clinical Outcomes in Rheumatoid Arthritis: A Systematic Review and Meta-Analysis
Source: Front Psychiatry. 2022 Mar 17;13:848427. doi: 10.3389/fpsyt.2022.848427 (PMC8968629; doi:10.3389/fpsyt.2022.848427)
Supplement: Supplementary file 2 [file Presentation_1.PDF]

Supplementary Appendix - Search algorithm

PubMed:

#1 ((rheumatoid arthritis[MeSH Terms]) OR (arthritis, rheumatoid[MeSH Terms])) OR (Rheumatoid Arthritis[Title/Abstract]) 155,922

#2 ((((((((((((((((((((((((((((((((((patient education[MeSH Terms]) OR (patient education as topic[MeSH Terms])) OR (Patient Education[Title/Abstract])) OR (Health education[Title/Abstract])) OR (health education[MeSH Terms])) OR (activity, educational[MeSH Terms])) OR (activities, educational[MeSH Terms])) OR (Education[Title/Abstract])) OR (Educational[Title/Abstract])) OR (Education of Patients[Title/Abstract])) OR (Educational Models[Title/Abstract])) OR (educational models[MeSH Terms])) OR (Education intervention[Title/Abstract])) OR (Education program[Title/Abstract])) OR (Instruction education[Title/Abstract])) OR (Education session[Title/Abstract])) OR (Education sevice[Title/Abstract])) OR (Educational Counseling[Title/Abstract])) OR (Health promotion[Title/Abstract])) OR (Lifestyle intervention[Title/Abstract])) OR (Psychoeducation[Title/Abstract])) OR (Rehabilitation Education[Title/Abstract])) OR (behavior therapy[MeSH Terms])) OR (cognitive behavior therapy[MeSH Terms])) OR (Lifestyle Changes[Title/Abstract])) OR (Health Behavior[Title/Abstract])) OR (Training Programs[Title/Abstract])) OR (training programs[MeSH Terms])) OR (Training Program[Title/Abstract])) OR (Workshop[Title/Abstract])) OR (Counseling[Title/Abstract])) OR (Telemedicine[Title/Abstract])) OR (Telehealth[Title/Abstract])) OR (telehealth[MeSH Terms])) OR (attitude to health[MeSH Terms]) 1,771,255

#3 #1 AND #2 4,815

#4 ((((((Randomized Controlled Trial[Publication Type]) OR (Clinical Trial[Publication Type])) OR (Randomly[Title/Abstract])) OR (Random[Title/Abstract])) OR (Randomized[Title/Abstract])) OR (Trial[Title/Abstract])) OR (Controlled[Title/Abstract]) 2,392,704

#5 #3 AND #4 958

Embase:

#1 'Rheumatoid Arthritis '/exp	222,209
#2 'rheumatoid arthritis':ab,ti,kw	174,261
#3 #1 OR #2	254,932
#4 'education'/exp	1,588,909
#5 'medical education'/exp	364,707
#6 'patient education'/exp	118,229
#7 'health education'/exp	352,113
#8 'patient education':ab,ti	29,604
#9 'health education':ab,ti	40,427
#10 'education':ab,ti	668,370
#11 'educational':ab,ti	230963
#12 'education of patients':ab,ti	1,865
#13 'educational models':ab,ti	395
#14 'educational activity':ab,ti	1,380
#15 'education intervention':ab,ti	3,130
#16 'education program':ab,ti	20,755
#17 'instruction education':ab,ti	18
#18 'education session':ab,ti	1,743
#19 'cognitive behavioral therapy'/exp	18,376
#20 'educational counseling':ab,ti	188
#21 'health promotion':ab,ti	38,167
#22 'lifestyle intervention':ab,ti	7,243
#23 'psychoeducation':ab,ti	6,297
#24 'rehabilitation education':ab,ti	205
#25 'behavior therapy':ab,ti	7,046
#26 'lifestyle changes':ab,ti	11,045

#27 'lifestyle modification':ab,ti 7,010

#28 'health behavior':ab,ti 9,306

#29 'workshop':ab,ti 44,295

#30 'telemedicine':ab,ti 19,637

#31 'telehealth':ab,ti 9,114

#32 #4 OR #5 OR #6 OR #7 OR #8 OR #9 OR #10 OR #11 OR #12 OR #13 OR #14 OR #15 OR #16 OR #17 OR #18 OR #19 OR #20 OR #21 OR #22 OR #23 OR #24 OR #25 OR #26 OR #27 OR #28 OR #29 OR #30 OR #31 1,974,790

#33 #3 AND #32 13,812

#34 'randomized controlled trial'/exp 684,593

#35 'randomized controlled trial (topic)'/exp 213,458

#36 #34 AND #35 894,012

#37 #33 AND #36 656

Cochrane Library:

#1 MeSH descriptor: [Arthritis, Rheumatoid] explode all trees 6,338

#2 ('Rheumatoid Arthritis'):ti,ab,kw 17,334

#3 #1 OR #2 17,640

#4 MeSH descriptor: [Education] explode all trees 34,477

#5 MeSH descriptor: [Early Intervention, Educational] explode all trees 522

#6 MeSH descriptor: [Patient Education as Topic] explode all trees 9,184

#7 MeSH descriptor: [Cognitive Behavioral Therapy] explode all trees 9,773

#8 ('Education'):ti,ab,kw 90,205

#9 ('Patient Education'):ti,ab,kw 46,888

#10 ('Health education'):ti,ab,kw 46,582

#11 ('Education intervention'):ti,ab,kw 56,066

#12 ('Educational Activity'):ti,ab,kw 20,371

#13 ('Education program'):ti,ab,kw 34,314

#14	('Instruction education'):ti,ab,kw	6,761
#15	('Psychoeducation'):ti,ab,kw	4,591
#16	('Rehabilitation Education'):ti,ab,kw	6,094
#17	('Cognitive behavioral therapy'):ti,ab,kw	25,294
#18	#4 OR #5 OR #6 OR #7 OR #8 OR #9 OR #10 OR #11 OR #12 OR #13 OR #14 OR #15 OR #16 OR #17	123,310
#19	#3 AND #18	621

Web of Science Database:

#1	TS = Rheumatoid Arthritis	255,152
#2	TI = Rheumatoid Arthritis	104,355
#3	AB = Rheumatoid Arthritis	100,488
#4	AK = Rheumatoid Arthritis	36,647
#5	#1 OR #2 OR #3 OR #4	255,152
#6	TS = Education	1,425,446
#7	TI = Education	253,985
#8	TS= Educational	435,621
#9	TI= Educational	56,407
#10	TS = Patient Education	367,840
#11	TI = Patient Education	14,612
#12	TS = Health education	597,574
#13	TI = Health education	25,407
#14	AB = Education	678,981
#15	AB = Educational	272,099
#16	AB = Patient Education	161,657
#17	AB = Health education	218,517
#18	AB = Education of Patients	161,059
#19	TI = Education of Patients	8,053

#20 AB = Educational Models	44,253
#21 AB = Educational Activity	41,195
#22 TI = Educational Activity	1,501
#23 TS = Education intervention	161,886
#24 TS = Education program	343,076
#25 TS = Instruction education	57,853
#26 TS = Education session	40,163
#27 TS = Educational Counseling	9,197
#28 TS = Lifestyle intervention	38,693
#29 TS = Psychoeducation	5,202
#30 TS = Rehabilitation Education	39,638
#31 TS = Cognitive behavioral therapy	196,488
#32 TI = Cognitive behavioral therapy	5,371
#33 TS = Lifestyle Changes	46,433
#34 TS = Telemedicine	50,191
#35 TS = Telehealth	12,007
#36 AB = Education intervention	86,857
#37 AB = Education program	170,913
#38 AB = Instruction education	15,605
#39 AB = Lifestyle intervention	29,540
#40 AB = Rehabilitation Education	10,895
#41 #6 OR #7 OR #8 OR #9 OR #10 OR #11 OR #12 OR #13 OR #14 OR #15 OR #16 OR #17 OR #18 OR #19 OR #20 OR #21 OR #22 OR #23 OR #24 OR #25 OR #26 OR #27 OR #28 OR #29 OR #30 OR #31 OR #32 OR #33 OR #34 OR #35 OR #36 OR #37 OR #38 OR #39 OR #40	1,870,022
#42 #5 AND #41	5,565
#43 TS = Randomized Controlled Trial	631,719
#44 TI = Randomized Controlled Trial	139,578
#45 TI = Randomized	313,540

#46 TI = Trial	477,204
#47 #43 OR #44 OR #45 OR #46	947,232
#48 #42 AND #47	708
